# RNA Interference Screen Identifies Abl Kinase and PDGFR Signaling in *Chlamydia trachomatis* Entry

**DOI:** 10.1371/journal.ppat.1000021

**Published:** 2008-03-07

**Authors:** Cherilyn A. Elwell, Alhaji Ceesay, Jung Hwa Kim, Daniel Kalman, Joanne N. Engel

**Affiliations:** 1 Department of Medicine, University of California San Francisco, San Francisco, California, United States of America; 2 Department of Pathology and Laboratory of Medicine, Emory University, Atlanta, Georgia, United States of America; 3 Department of Microbiology and Immunology, University of California San Francisco, San Francisco, California, United States of America; Pasteur Institute, France

## Abstract

To elucidate the mechanisms involved in early events in *Chlamydia trachomatis* infection, we conducted a large scale unbiased RNA interference screen in *Drosophila melanogaster* S2 cells. This allowed identification of candidate host factors in a simple non-redundant, genetically tractable system. From a library of 7,216 double stranded RNAs (dsRNA), we identified ∼226 host genes, including two tyrosine kinases, Abelson (Abl) kinase and PDGF- and VEGF-receptor related (Pvr), a homolog of the Platelet-derived growth factor receptor (PDGFR). We further examined the role of these two kinases in *C. trachomatis* binding and internalization into mammalian cells. Both kinases are phosphorylated upon infection and recruited to the site of bacterial attachment, but their roles in the infectious process are distinct. We provide evidence that PDGFRβ may function as a receptor, as inhibition of PDGFRβ by RNA interference or by PDGFRβ neutralizing antibodies significantly reduces bacterial binding, whereas depletion of Abl kinase has no effect on binding. Bacterial internalization can occur through activation of PDGFRβ or through independent activation of Abl kinase, culminating in phosphorylation of the Rac guanine nucleotide exchange factor (GEF), Vav2, and two actin nucleators, WAVE2 and Cortactin. Finally, we show that TARP, a bacterial type III secreted actin nucleator implicated in entry, is a target of Abl kinase. Together, our results demonstrate that PDGFRβ and Abl kinases function redundantly to promote efficient uptake of this obligate intracellular parasite.

## Author Summary

*Chlamydia trachomatis* infections are a worldwide problem; they are the leading cause of preventable blindness in developing nations and the most common cause of sexually transmitted disease in the Western world. Binding and entry into host cells are critical steps to the pathogenesis of this obligate intracellular parasite; however little is known regarding the mechanism of these processes. In this work, we describe a large scale RNA interference screen to identify host factors essential for early steps in *C. trachomatis* infection. We discover that the Platelet Derived Growth Factor Receptor β (PDGFRβ) can function as a receptor for *C. trachomatis,* and that activation of both PDGFRβ and Abl kinase signaling pathways by *C. trachomatis* leads to phosphorylation of a Rac guanine nucleotide exchange factor, Vav2, and several actin nucleators, including WAVE2, Cortactin, and TARP, a *Chlamydia* type III secreted effector. Our work suggests a model of redundant activation of PDGFRβ and Abl kinase upon *C. trachomatis* binding that culminates in cytoskeletal rearrangements that modulate efficient uptake of this obligate intracellular parasite.

## Introduction

*Chlamydia* species cause a wide range of diseases in humans, including sexually transmitted, ocular, and respiratory tract infections (reviewed in [1]). Chronic *Chlamydia* infections can result in female infertility, blindness, and possibly atherosclerosis [2]. Despite the broad spectrum of *chlamydial* diseases, all *Chlamydia* species share a common strategy that allows this obligate intracellular parasite to survive within the host cell [3]. Infection is initiated by binding and internalization of the extracellular infectious form, the elementary body (EB), which is small (0.3 µm) and metabolically inactive, into target host cells. EBs enter into a membrane-bound compartment, which quickly dissociates from the endocytic pathway and avoids phagolysosomal fusion [4]. Within a few hours, EBs differentiate into larger (1 µm) reticulate bodies (RBs) that represent the metabolically active, replicative form. The RBs replicate by binary fission within the enlarging inclusion over a 48–72 hour time period, and then undergo a second differentiation process back to infectious EBs, which are released from host cells.

The mechanisms involved in binding and uptake, including the identity of the bacterial ligand and the host cell receptor, are incompletely defined. The fact that chlamydiae can productively infect most cultured cells suggests that the receptor(s) may be widespread, that chlamydiae utilize multiple means of entry, and/or that these organisms inject their own receptor, possibly by type III secretion. For some species and serovars, including the more invasive Lymphogranuloma Venereum (LGV) serovar L2, heparan sulfate has been suggested to act as a bridging molecule for a relatively weak and reversible interaction [5]–[8] that is followed by a stronger, more specific binding to an unidentified secondary receptor [9],[10]. Internalization of *Chlamydia* is accompanied by induction of microvilli-like structures over a large portion of the host cell in a process that is dependent upon actin polymerization [11]. Indeed, actin reorganization is observed at the sites of *Chlamydia* entry [12],[13], and inhibition of actin polymerization blocks entry [11]. The Rho family GTPase, Rac, is activated upon infection and is required for *C. trachomatis* entry while both Rac and Cdc42 appear to be required for *C. caviae* entry [12],[13].

The molecules involved in Rac activation and in subsequent actin rearrangements necessary for *C. trachomatis* entry are unknown. Tyrosine phosphorylation of several unidentified proteins has been observed early in infection, and tyrosine phosphorylated proteins have been shown to accumulate around EBs at the entry site for several *Chlamydia* species [14]–[18]. In the case of *C. trachomatis*, Translocated actin recruiting phosphoprotein (TARP), a type III secreted bacterial protein, is rapidly tyrosine phosphorylated upon infection and is associated with actin recruitment [17]. While TARP is encoded in all *Chlamydia* species examined thus far, only *C. trachomatis* TARP orthologs possess several tyrosine-rich tandem repeats of approximately 50 amino acids in length [17]. Studies utilizing ectopic expression of *C. trachomatis* TARP in mammalian cells reveal that phosphorylation occurs within this repeat region in the N-terminus and that the C-terminal domain of TARP is essential for actin recruitment [16]. In addition, a recent report reveals that TARP can nucleate actin in vitro [19]. Based on these observations, TARP has been proposed to play a role in bacterial internalization by modulating cytoskeletal reorganization at the site of entry. The tyrosine kinase that phosphorylates TARP has not been identified, however it is believed to be of host origin since TARP is phosphorylated upon transfection in mammalian cells or when delivered into host cells by the *Yersinia* type III secretion system [16],[17].

The study of *Chlamydia* pathogenesis has been hampered by the inability to carry out traditional genetics. Genetically tractable organisms, including *Drosophila melanogaster,* provide an attractive alternate avenue of exploration. The ease and availability of using RNA interference (RNAi) to inactivate gene expression and the lack of redundancy in the genome compared to mammals affords the opportunity to uncover complicated or redundant signaling pathways that might otherwise be overlooked. In this paper, we describe a high throughput RNAi based screen to identify host factors required for early steps in *C. trachomatis* infection. We have focused on a subset of candidates known to be important for Rac-dependent cytoskeletal rearrangements, including PDGF- and VEGF-receptor related (Pvr; a homolog of the Platelet-derived growth factor receptor (PDGFR)), Abelson (Abl) kinase, Vav, WAVE, and Cortactin [20]–[26]. Using genetic, chemical, and biochemical approaches, we examined the role of these signaling molecules in mammalian infections. We demonstrate that PDGFRβ functions as a receptor for *C. trachomatis*. Once bound, bacterial internalization can occur either through activation of PDGFRβ or through independent activation of Abl kinase. Activation of these kinases leads to phosphorylation of the Rac guanine nucleotide exchange factor (GEF) Vav2, and several actin nucleators, including WAVE2, Cortactin, and TARP, that likely promote efficient uptake of this obligate intracellular parasite.

## Results

### RNAi screen to identify host proteins required for *C. trachomatis* infection

We have previously shown that *C. trachomatis* infection of *D. melanogaster* S2 cells recapitulates early steps in the interaction of *C. trachomatis* with mammalian cells, including binding, entry, and escape from phagolysomal fusion [27]. Later steps, such as bacterial replication or release from S2 cells do not occur because of the very limited replication of *C. trachomatis* below 30°C and the inability of S2 cells to survive above 30°C [27]. Here, we used a library of 7,216 double stranded RNAs (dsRNA) representing most of the phylogenetically conserved genes of *D. melanogaster* [28] to perform an RNAi screen in S2 cells to identify host gene products important for early stages of *C. trachomatis* infection, up to and including inclusion formation. For screening, S2 cells were incubated with each individual dsRNA for 4 days and then infected with *C. trachomatis* strain L2 for 48 hours. Cells were then replated onto 96 well glass bottom plates, fixed, and stained with a Fluorescein isothiocyanate (FITC)-conjugated antibody that recognizes the *Chlamydia* major outer membrane protein (MOMP) to visualize inclusions, and counterstained with Evan's blue to visualize cells. We performed a visual screen to identify changes in the efficiency of inclusion formation, alterations in inclusion morphology, and decreased host cell viability.

The primary screen identified 862 putative host genes required for early steps in *C. trachomatis* infections. A large number of these genes appeared to be involved in general host cell processes, such as gene expression and protein degradation. Indeed, many of these genes have been identified in other screens for microbial uptake [29],[30]. Since depletion of many of these genes would be predicted to have pleiotropic affects that would likely indirectly affect *C. trachomatis* infection, these genes were not further analyzed.

**Figure 1:**
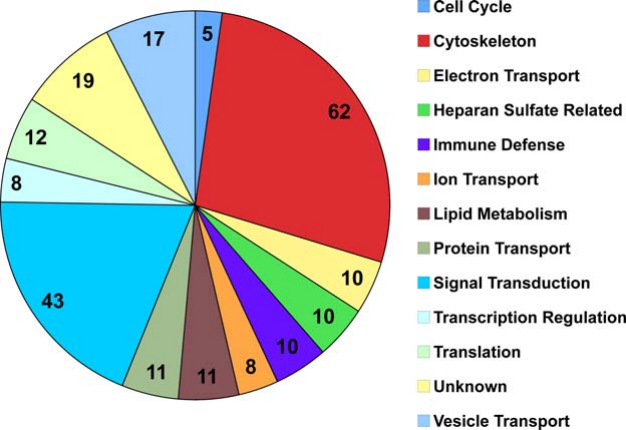
Categories of host genes identified in RNAi screen. Number of genes in each class is indicated.

We retested 360 dsRNAs and identified approximately 226 host gene products that likely participate in distinct stages of the *C. trachomatis* developmental cycle, including binding, entry, trafficking, inclusion formation, inhibition of phagolysosomal fusion, survival, or modulation of host signaling pathways. The gene products that affected *C. trachomatis* inclusion formation identified in the screen can be divided into the following categories: Cell Cycle, Cytoskeleton, Electron Transport, Heparan Sulfate Metabolism, Immune Defense, Ion Transport, Lipid Metabolism, Protein Transport, Signal Transduction, Transcription Regulaton, Translation, and Vesicle Transport (Figure 1 and Table S1). In many cases, we identified multiple subunits of known protein complexes or multiple components of known biochemical pathways. Some of the proteins have been previously implicated in *C. trachomatis* infection, further supporting their role in *C. trachomatis* infection and also validating our approach. These included (1) Lace, the *Drosophila* homolog of Spt-1 (sphingosine palmitoyl transferase), the rate limiting enzyme in sphingolipid biosynthesis, which is necessary for intracellular survival [31]; (2) *Rac*, which is required for *C. trachomatis* internalization [12]; (3) enzymes involved in heparan sulfate metabolism, which is consistent with the known requirement for heparan sulfate in binding [5]–[8]; and (4) several Rabs and Rab-related proteins, which have been shown to localize to the vacuole [32]–[35]. The identification of factors known to be involved in *C. trachomatis* infection gave us confidence that the novel candidates could be playing a previously unrecognized role in infection by *C. trachomatis*.

We were particularly interested in two tyrosine kinases, PDGFR and Abl kinase, because (1) several components of the signal transduction pathways involving PDGFR and Abl kinase [20]–[26],[36],[37] were identified in our screen, including Abi, WAVE, Nap, Crk, Vav, Arp2/3, and Cortactin, (2) both kinases are involved in Rac-dependent cytoskeletal rearrangements, processes known to be required for *C. trachomatis* entry [12], and because (3) *C. trachomatis* entry is associated with accumulation of tyrosine phosphorylated proteins at the site of entry, including the bacterially-encoded TARP. We further examined the role of these kinases in *C. trachomatis* infection of mammalian cells.

### Platelet-derived growth factor receptor β is activated upon infection and recruited to the site of EB entry

**Figure 2:**
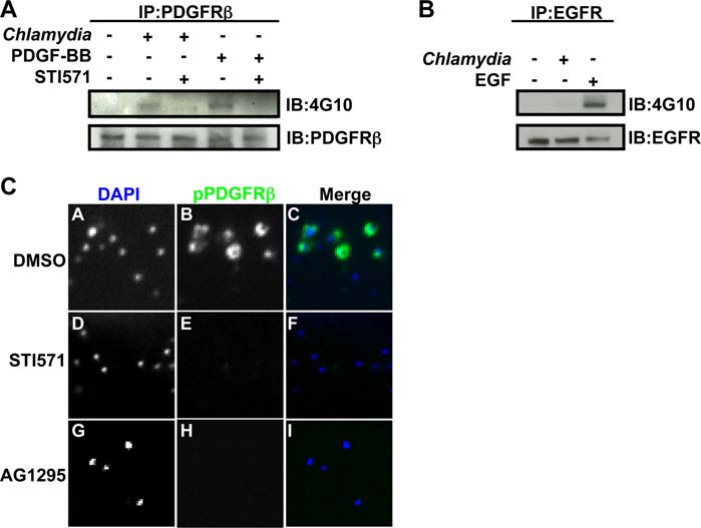
*C. trachomatis* infection induces phosphorylation of PDGFRβ and recruitment of phospho-PDGFRβ to the site of entry. (A) PDGFRβ was immunoprecipitated from *C. trachomatis* infected HeLa cells at 1 hpi in the absence or presence of STI571 and immunoblotted with 4G10 antibody. As a positive control, cells were stimulated with 100 ng/ml PDGF-BB. (B) EGFR was immunoprecipitated from *C. trachomatis* infected HeLa cells at 1 hpi and immunoblotted with 4G10 antibody. As a positive control, cells were stimulated with 100 ng/ml EGF. (C) *C. trachomatis* infected cells incubated in the absence (panels A–C) or presence of STI571 (panels D–F) or AG1295 (panels G–I) were fixed and stained with anti-phospho-PDGFRβ antibody (green in merge). Bacteria and host DNA were detected using DAPI (blue in merge). The exposure time for each filter of all images was identical.

Members of the PDGF receptor family are receptor tyrosine kinases and consist of two receptor forms, PDGFRα and PDGFRβ, that form homo or heterodimers upon binding of the growth factor receptor ligand, PDGF [38]. These receptors are important for cell proliferation and cell migration [38]. Receptor dimerization upon ligand binding stimulates the intrinsic tyrosine kinase activity of the receptors, leading to receptor autophosphorylation as well as recruitment and tyrosine phosphorylation of various downstream effectors [38]. To determine whether PDGFRβ is activated upon infection, HeLa cells were infected with L2 for 1 hour, and lysates were immunoprecipitated with anti-PDGFRβ antibodies followed by immunoblotting with the anti-phosphotyrosine antibody, 4G10. As a positive control for receptor phosphorylation and activation, a parallel set of cells was treated with PDGF-BB. As shown in Figure 2A, PDGFRβ was phosphorylated upon infection with L2. This phosphorylation was blocked when cells were incubated prior to and during infection with two chemically distinct inhibitors of PDGFR activity, STI571 [39],[40], (Figure 2A), or AG1295 [41] (data not shown). Whereas STI571 also inhibits Abl family kinases, AG1295 is specific for PDGFR. Pretreatment of EBs with STI571 or AG1295 did not affect their viability, as evidenced by their ability to form normal inclusions in HeLa cells (data not shown). L2 did not induce phosphorylation of Epidermal Growth Factor Receptor (EGFR; Figure 2B), suggesting there is specificity for PDGFRβ.

We confirmed these results by examining whether catalytically activated PDGFRβ was recruited to EBs during entry using immunofluorescence microscopy (IF) with an antibody that recognizes phosphorylated (activated) PDGFRβ. As shown in Figure 2C, phosphorylated PDGFRβ was recruited to most but not all bound EBs; this phosphorylation was inhibited by STI571 and AG1295 (Figure 2C). We could not detect recruitment of phosphorylated EGFR to EBs (data not shown).

### PDGFRβ contributes to *C. trachomatis* L2 binding

**Figure 3:**
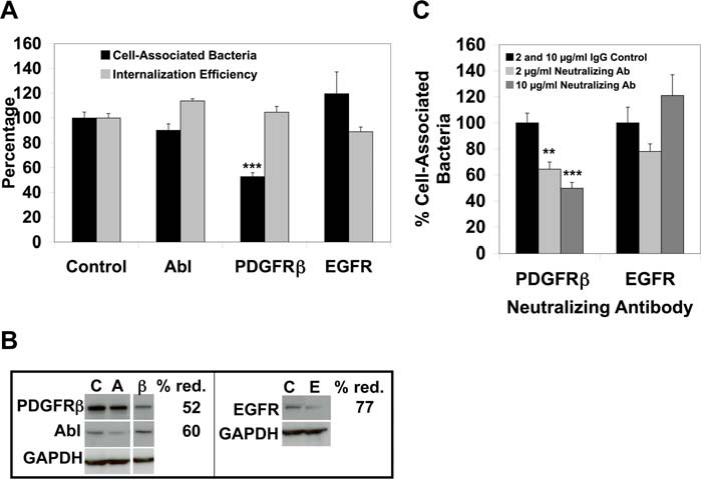
PDGFRβ contributes to *C. trachomatis* binding. (A) HeLa cells were transfected with control siRNA or siRNA targeting Abl kinase, PDGFRβ, or EGFR and subsequently infected with *C. trachomatis* for 1 hour. Cells were fixed and analyzed by inside out staining for the total cell-associated bacteria (black bars) and the internalization efficiency (light grey bars). The data are expressed as the percentage of cell-associated bacteria (which includes internalized and surface-associated bacteria) or as the internalization efficiency (the percentage of internalized EBs/total cell-associated EBs). In each case, the data are normalized to control siRNA. ***p<0.001 for PDGFRβ RNAi-treated cells compared to control RNAi-treated cells (ANOVA). The percentage of cell-associated bacteria was significantly decreased only in cells depleted of PDGFRβ, but not Abl kinase or EGFR. (B) Western blot analysis of siRNA-treated samples. C, control; A, Abl; β, PDGFRβ; E, EGFR. Control, Abl and PDGFRβ samples were run on the same blot. GAPDH was used as a loading control. The efficiency of protein depletion compared to control siRNA-treated cells is indicated to the right of each panel. (C) HeLa cells were pretreated with control IgG or a neutralizing antibody to PDGFRβ or EGFR at the indicated concentrations, subsequently infected with *C. trachomatis* for 1 hour, and analyzed for total cell-associated EBs. Data for PDGFRβ or EGFR neutralizing antibody was normalized to the same concentration of each isotype matched IgG control and set at 100%. ***p<0.001 or **p<0.01 for cells pretreated with PDGFRβ neutralizing antibody compared to the same concentration of isotype matched IgG control (ANOVA). The percentage of cell-associated bacteria was significantly decreased only in cells treated with a neutralizing antibody to PDGFRβ but not EGFR. The small decrease in binding observed upon pretreatment with 2 ug/ml of EGFR neutralizing antibody is not statistically significant.

Since PDGFR is a transmembrane receptor, we investigated the possibility that PDGFRβ could serve as a receptor or coreceptor for *C. trachomatis.* Using RNAi, we depleted HeLa cells of PDGFRβ EGFR, or Abl kinase for 48 hrs. Cells were then infected with *C. trachomatis* for 1 hour and analyzed by inside out staining as described in Materials and Methods. The efficiency of protein depletion was determined by Western blot analysis (Figure 3B). We first determined the total number of cell-associated bacteria (which includes internalized and surface-associated bacteria) for each treatment, and expressed this number as percent of cell-associated bacteria normalized to the control small interfering RNA (siRNA) sample. PDGFRβ depletion resulted in a 50% reduction in cell-associated bacteria, whereas depletion of EGFR or Abl kinase had no effect (Figure 3A). We then assessed internalization efficiency in the same cells by determining the percentage of internalized EBs/total cell-associated EBs. PDGFRβ EGFR, and Abl kinase depletion showed no effect on internalization efficiency (Figure 3A). Together, these data show that depletion of PDGFRβ diminishes host-cell association but not internalization efficiency of bacteria, suggesting that PDGFRβ is important for binding to host cells. Its role in internalization will be discussed later.

We further confirmed a role for PDGFRβ in binding by using a neutralizing antibody to PDGFRβ. A dose-dependent decrease in cell-associated bacteria was observed when cells were preincubated with a PDGFRβ neutralizing antibody, whereas an EGFR neutralizing antibody had no significant effect (Figure 3C). Both PDGFRβ and EGFR neutralizing antibodies showed no effect on the internalization efficiency of *C. trachomatis* into cells (data not shown). These results indicate that PDGFRβ mediates binding of *C. trachomatis* L2 to host cells. However, as none of the PDGFRβ-directed treatments completely abolished host-cell association, other cellular receptors may also contribute to *C. trachomatis* binding.

### Abl kinase is activated and recruited to EBs

**Figure 4:**
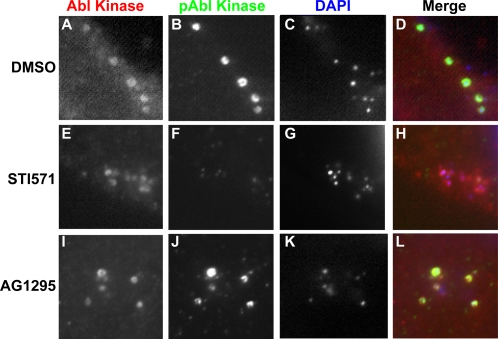
Activated Abl kinase is recruited to EBs during entry. HeLa cells were pretreated with DMSO (panels A–D), STI571 (panels E–H), or AG1295 (panels I–L) for 1 hour and subsequently infected with *C. trachomatis* in the presence of carrier or the indicated drug. The cells were fixed and stained with antibody that recognizes endogenous Abl kinase (panels A, E, and I; red in merge) and an antibody that recognizes phospho-Abl Y412 (panels B, F, and J; green in merge). Bacteria and host DNA were detected using DAPI (panels C, G, and K; blue in merge). The merged panels are shown on the right (panels D, H, L). The exposure time for each filter of all images was identical. PDGFR activity is not required for recruitment and activation of Abl kinase.

The Abl kinase family, which includes Abl and Arg, are nonreceptor tyrosine kinases comprised of a Src homology 2 (SH2) domain, a Src homology 3 (SH3) domain, an activation domain that can undergo autophosphorylation, and actin binding domains (reviewed in [18]). Abl kinases are involved in many important mammalian processes, including cell cycle regulation, apoptosis, response to DNA damage, and Rac-dependent cytoskeletal dynamics [18], [42]–[45]. To determine whether Abl kinase is activated upon infection, HeLa cells infected with *C. trachomatis* for 1 hour were fixed and stained with an antibody that specifically recognizes Abl when it is phosphorylated on Y412 in the activation loop domain; this phosphorylation is an indicator of its catalytic activation [46]–[48]. Abl kinase (Figure 4, panels A–D) and the closely related Arg kinase (data not shown) were activated and recruited to most cell-associated EBs. Bacterial infection also resulted in a significant increase in the phosphorylation of CrkII, a substrate of Abl kinase (Figure S4), further supporting that Abl kinase was activated upon EB binding. Moreover, phosphorylated CrkII was recruited to EBs in infected HeLa and wild type 3T3 cells (data not shown), whereas no recruitment of phosphorylated CrkII was observed in 3T3 cells derived from mice lacking both Abl and Arg kinase (data not shown) [49]. Pretreatment of cells with STI571 blocked *C. trachomatis*-induced Abl phosphorylation, without affecting recruitment of Abl kinase to EBs (Figure 4, panels E–H and Figure S1; 89% reduction; ***p<0.001). We could not detect changes in Abl kinase phosphorylation by immunoblot analysis of infected lysates using anti-Y412 antibody, presumably because Abl activation occurs locally at the site of EB entry and represents only a small percentage of the total Abl in the cell. In addition, activated Abl has been shown to undergo rapid ubiquitin-mediated degradation by the proteasome [50],[51].

It has been reported that Abl activation occurs by both PDGFR-dependent and independent pathways [44]. We examined whether *C. trachomatis* stimulation of Abl kinase activty was dependent on PDGFR activation. Whereas STI571 inhibited *C. trachomatis-*induced recruitment of phosphorylated Abl kinase, AG1295, a specific inhibitor of PDGFR, had no effect (Figure 4, panels I–L, and Figure S1). Together, these results indicate that while PDGFR can function as a receptor for *C. trachomatis* and is activated by bacterial binding, activation of Abl kinase by *C. trachomatis* can occur independently of PDGFR activation.

### Abl kinases are required for tyrosine phosphorylation of proteins associated with EBs at the site of entry

**Figure 5:**
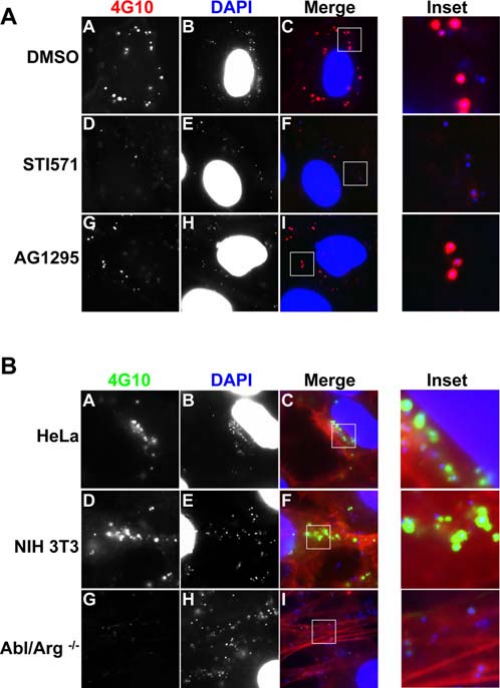
Abl Kinase is necessary for tyrosine phosphorylation of proteins associated with EBs. (A) HeLa cells were pretreated with DMSO (panels A–C), STI571 (panels D–F), or AG1295 (panels G–I) for 1 hr, infected with *C. trachomatis,* and phospho-tyrosine-associated proteins detected by staining with 4G10 (panels A, D, and G; red in merge). Bacteria and host DNA were detected using DAPI (panels B, E, and H; blue in merge). The exposure time for each filter of all images was identical. STI571 but not AG1295 inhibits EB-associated tyrosine phosphorylation. (B) HeLa, NIH 3T3, and Abl/Arg^−/−^ cells were infected with *C. trachomatis* for 1 hour and then stained with 4G10 (panels A, D, G; green in merge). Actin was stained with phalloidin (panels C, F, I; red in merge). Bacteria and host DNA were detected using DAPI (panels B, E, H; blue in merge). The exposure time for each filter of all images was identical. EB-associated tyrosine phosphorylation is diminished in Abl/Arg^−/−^ cells.

Several groups have reported that EB entry is associated with recruitment of tyrosine phosphorylated proteins [15],[16],[52],[53]. We used pharmacologic and genetic approaches to determine whether PDGFR and Abl family kinases contributed to the tyrosine phosphorylation events associated with EBs during entry. Pretreatment of *C. trachomatis*-infected HeLa cells with STI571 diminished association of phospho-tyrosine-containing proteins with EBs by 90%, as assessed by IF using the 4G10 antibody (Figure 5A, panels D–F, and see Figure S1 for quantitation, ***p<0.001). In contrast, AG1295 did not inhibit *C. trachomatis*-induced tyrosine phosphorylation of proteins associated with EBs (Figure 5A, panels G–I, and Figure S1).

We confirmed the role of Abl kinases in phosphorylating EB-associated proteins by examining tyrosine phosphorylation of *C. trachomatis*-infected Abl/Arg^−/−^ cells or Abl siRNA-depleted HeLa cells. We observed a significant reduction in the number of EBs associated with tyrosine phosphorylated proteins in the Abl/Arg^−/−^ cells as compared to the 3T3 control cells (Figure 5B and Figure S2; 75% reduction, ***p<0.001). Similar results were observed when Abl kinase levels were depleted 85% by siRNA (Figure S2; 60% reduction in EB-associated phospho-tyrosine proteins compared to control siRNA-treated cells, ***p<0.001). This result is similar to 3T3 cells (Figure S2) and control RNAi-treated HeLa cells exposed to STI571 (Figure S2). Together, these results suggest that Abl kinase and/or Abl kinase targets comprise the majority of EB-associated tyrosine phosphorylated proteins. Tyrosine phosphorylation was not entirely abolished in the Abl/Arg^−/−^ cells, however the residual phosphorylation was less intense (Figure 5B). These data indicate that there is also an Abl kinase-independent pathway of tyrosine phosphorylation that most likely involves PDGFRβ.

To determine whether Abl kinase was sufficient for tyrosine phosphorylation of EB-associated proteins, we examined phosphorylation in Abl/Arg^−/−^ cells transiently transfected with Hemaglutinin (HA)-tagged Abl kinase. Expression of HA-Abl in Abl/Arg^−/−^ cells restored tyrosine phosphorylation to similar levels as those observed in 3T3 cells (Figure S3), whereas expression of a control protein, enhanced green fluorescent protein (EGFP), had no affect on tyrosine phosphorylation. Taken together, these results indicate that Abl kinase is necessary and sufficient for tyrosine phosphorylation of proteins at the site of EB invasion.

### Abl and PDGFR kinases function redundantly in *C. trachomatis* entry

To determine whether activation of Abl kinase and/or PDGFRβ was necessary for *C. trachomatis* entry, we used three complementary approaches to functionally inhibit PDGFR and Abl kinase activities either individually or in combination: 1) pharmacological inhibitors of PDGFR and Abl, 2) Abl/Arg^−/−^ cells, and 3) siRNA-treated HeLa cells. For these experiments, cells were infected with *C. trachomatis* and then analyzed by inside out staining to determine the total cell-associated EBs and internalization efficiency. The percentage of cell-associated bacteria was not significantly decreased with drug treatment (data not shown), in Abl/Arg^−/−^ cells (data not shown), or when Abl kinase was depleted by siRNA (Figure 3).

**Figure 6:**
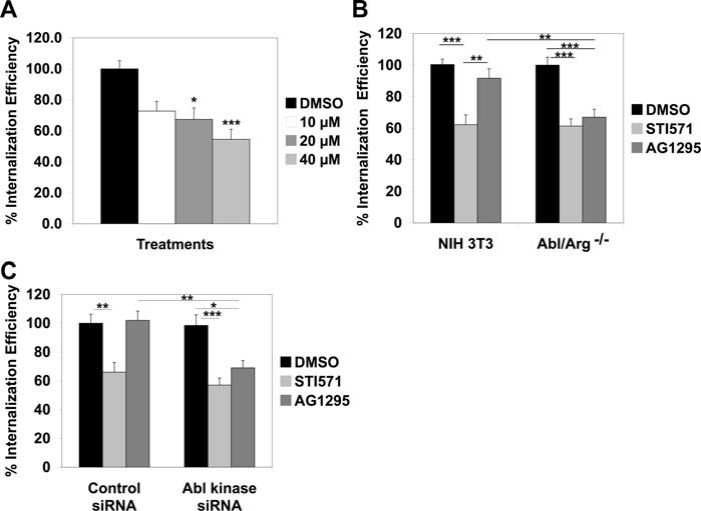
Abl kinase and PDGFR function redundantly in *C. trachomatis* entry. (A) HeLa cells were pretreated with DMSO or the indicated concentration of STI571 for 1 hr and subsequently infected with *C. trachomatis* in the presence of DMSO or STI571. Cells were fixed, stained, and analyzed for *C. trachomatis* internalization efficiency as described in Materials and Methods. Values (mean±s.e.m.) are shown as percentage of internalized EBs/total cell-associated EBs and normalized to DMSO-treated-HeLa cells. Data are from at least 3 independent experiments. *p<0.05 for cells treated with 20 µM STI571 compared to DMSO-treated cells. ***p<0.001 for cells with 40 µM STI571 compared to DMSO-treated cells (ANOVA). (B) NIH 3T3 or Abl/Arg^−/−^ cells were pretreated with DMSO, STI571, or AG1295 for 1 hour and subsequently infected with *C. trachomatis* in the presence of carrier or drug. Cells were fixed, stained, and analyzed for *Chlamydia* internalization efficiency as described in Materials and Methods. Values (mean±s.e.m.) are shown as percentage of internalized EBs/total EBs normalized relative to the DMSO control for each cell type. Data are from at least 3 independent experiments. There was no significant decrease in internalization efficiency in DMSO-treated Abl/Arg^−/−^ cells or AG1295-treated 3T3 cells compared to DMSO-treated 3T3; however, there was a significant reduction in internalization efficiency when PDGFR activity was inhibited in Abl/Arg^−/−^ cells and in STI571-treated cells. ***p<0.001 and **p<0.01 (ANOVA). (C) Control or Abl siRNA-treated HeLa cells were pretreated with DMSO, STI571, or AG1295 for 1 hour and subsequently infected with *C.trachomatis* in the presence of carrier or drug. Cells were fixed, stained, and analyzed for *C. trachomatis* internalization efficiency as described in Materials and Methods. Values (mean±s.e.m.) are shown as percentage of internalized EBs/total cell-associated EBs. All samples are normalized to DMSO-treated control siRNA cells. Data are from two independent experiments. There was no significant decrease in internalization efficiency in DMSO-treated Abl siRNA-treated cells or AG1295-treated control siRNA cells compared to DMSO-treated control siRNA-treated cells; however, there was a significant reduction in internalization efficiency when PDGFR activity was inhibited in Abl siRNA-treated cells and in STI571-treated cells. ***p<0.001, **p<0.01, and *p<0.05 (ANOVA).

Pretreatment of HeLa cells with STI571 decreased *C. trachomatis* internalization efficiency in a dose-dependent manner, with maximal inhibition of approximately 40–50% compared to DMSO-treated cells (Figure 6A). These results correlated with a dose-dependent inhibition of Abl kinase activity with maximal inhibition at 40 µM STI571 (Figure S4). As STI571 is highly protein bound [54] and these experiments were of necessity carried out in the presence of serum, these circumstances may explain why higher doses of STI571 (ie 40 µm) were required for maximal inhibition of Abl kinase activity and bacterial internalization.

We next examined *C. trachomatis* internalization efficiency in Abl/Arg^−/−^ cells or HeLa cells depleted of Abl by RNAi. No reduction in internalization efficiency was observed in the Abl/Arg^−/−^ cells compared to DMSO-treated 3T3 cells (Figure 6B). Similarly, infection of HeLa cells in which Abl kinase levels were decreased 85% by RNAi (Figure S2) did not affect *C. trachomatis* internalization efficiency (Figure 6C). To assess the contribution of PDGFR signaling during *C. trachomatis* entry, AG1295 was added to control siRNA-treated HeLa cells or 3T3 cells. As shown in Figure 6B and 6C, AG1295 had no significant effect on entry in control siRNA-treated cells. However, addition of AG1295 to Abl kinase depleted or to Abl/Arg^−/−^ cells diminished entry to a level similar to what was observed with STI571 treatment (Figure 6B and 6C). These results indicate that Abl and PDGFR kinases function redundantly in entry. Since entry was not completely abolished when Abl and PDGFR kinases were inhibited, other kinases likely contribute to bacterial entry.

**Figure 7:**
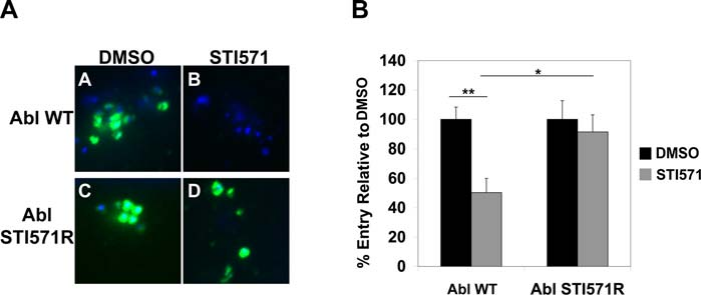
Abl kinase is sufficient for *C. trachomatis* entry. HeLa cells were transfected with Abl kinase (Abl WT) or an STI571 resistant allele of Abl kinase (Abl STI571R), treated with STI571, and then infected with *C. trachomatis* for 1 hour. (A) Cells were fixed and stained with an antibody that recognizes phosphorylated Abl kinase (green) and DAPI to identify bacteria (blue). The exposure time for each filter of all images was identical. Transfection of Abl STI571R (panel D) but not wild type Abl (panel B) permits recruitment of phospho-Abl in the presence of STI571. (B) A parallel set of cells were fixed, stained, and analyzed for *C. trachomatis* internalization efficiency as described in Materials and Methods. The internalization efficency (mean±s.e.m.) is the percentage of internalized EBs/total cell-associated EBs. Data are representative of three independent experiments. Samples are normalized to DMSO-treated cells. Expression of Abl STI571R permits internalization in the presence of STI571. **p<0.01 for STI571-treated Abl WT cells compared to DMSO-treated Abl WT cells. *p<0.05 for STI571-treated Abl WT cells compared to STI571-treated Abl STI571R cells (ANOVA).

We determined whether Abl kinase was sufficient for entry by examining the ability of a mutant version of Abl kinase that is resistant to STI571 (Abl STI571R) to support *C. trachomatis* internalization in the presence of STI571. This construct has a mutation in the ATP-binding domain of Abl, T315I, which interferes with STI571 binding to the active site [55]. We infected HeLa cells expressing wild type Abl kinase (Abl WT) or Abl STI571R in the presence or absence of STI571 and measured internalization efficiency. A parallel set of cells was assessed for *C. trachomatis*-induced activation of Abl kinase. As shown in Figure 7A, STI571 blocked recruitment of phosphorylated Abl kinase to the site of EB binding in cells transfected with Abl WT but not in cells expressing Abl STI571R, confirming that this allele is resistant to STI571. Addition of STI571 to cells transfected with Abl WT resulted in approximately a 50% decrease in internalization efficiency, similar to its observed inhibition of *C. trachomatis* entry in cells expressing endogenous Abl (Figure 6). In contrast, transfection with Abl STI571R permitted bacterial internalization in the presence of STI571 (Figure 7B). These results indicate that Abl kinase is sufficient to support *C. trachomatis* entry.

### Abl kinase and PDGFR signal to overlapping downstream effectors

In our S2 screen, we identified several tyrosine-phosphorylated proteins that are important for Rac-dependent actin remodeling and are known to be downstream of Abl kinase and PDGFR [20]–[26]. These include WAVE, Abelson interactor protein (Abi), Vav, and Cortactin. Interestingly, WAVE2 and Cortactin, activators of the Arp2/3 complex that nucleates actin, have been previously shown to colocalize with EBs [15],[19],[56]. Vav has not been previously implicated in *C. trachomatis* entry. Vav proteins have a number of domains, including pleckstrin homology (PH), DBL-homology (DH), SH2, SH3, and proline-rich domains, that allow them to function as guanine nucleotide exchange factors for Rac/Rho GTPases as well as scaffolding proteins (reviewed in [57]). Tyrosine phosphorylation of WAVE, Vav, and Cortactin closely correlates with their activation. Abl kinase has been shown to phosphorylate both WAVE2 and Cortactin upon PDGF stimulation [24]–[26] while Vav1 is a substrate of the oncogenic chimeric Bcr-Abl protein [58]. Vav2, the more ubiquitiously expressed form of Vav, has been shown to be a substrate of PDGFR [23]. Using immunoblot analysis in conjunction with Abl depletion and/or the PDGFR inhibitor, AG1295, we determined whether *C. trachomatis* infection induced phosphorylation of WAVE2, Vav2, and Cortactin in an Abl kinase and/or PDGFR-dependent manner in mammalian cells.

**Figure 8:**
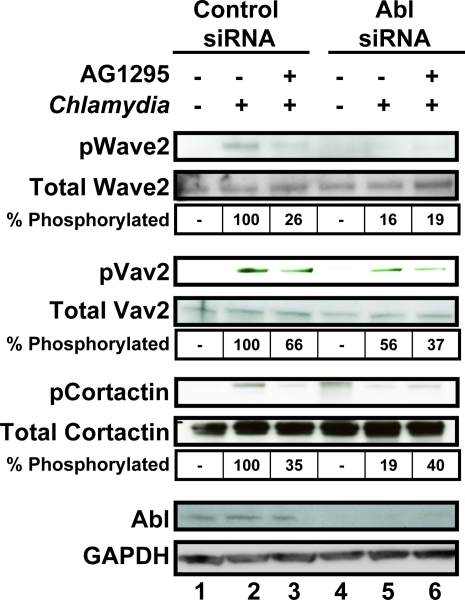
Abl kinase and PDGFR signal to overlapping downstream effectors. HeLa cells were transfected with control or Abl kinase siRNA for 48 hours, treated with DMSO or AG1295 for 1 hour, and then subsequently infected with *C. trachomatis* for 1 hour. WAVE2 and Cortactin were immunoprecipitated from lysates and subjected to western blot analysis with 4G10 to assess phosphorylation. Blots were reprobed for total protein amounts. Lysates from the same set of samples were probed with anti-pVav2, total Vav2, total Abl, and GAPDH (loading control) antibodies. The percentage of phosphorylated protein compared to total protein was quantified by densitometry analysis and normalized relative to *C. trachomatis*-infected samples. Immunoblots shown are representative of three independent experiments. *C. trachomatis* induces phosphorylation of WAVE2, Vav2, and Cortactin (lane 2). Inhibition of PDGFR or Abl signaling decreases WAVE2, Vav2, and Cortactin phosphorylation.

We first tested whether WAVE2, Vav2, and Cortactin were tyrosine phosphorylated upon *C. trachomatis* infection. L2 infection of HeLa cells induced tyrosine phosphorylation of WAVE2, Vav2, and Cortactin at 1 hour post infection (Figure 8, lane 2). In addition, phospho-Vav2 and phospho-Cortactin were recruited to the site of EB entry (data not shown). Tyrosine phosphorylation of WAVE2 and Cortactin was diminished (at least 3-fold) when Abl kinase was depleted (Figure 8, lane 5) or when PDGFR was inhibited by AG1295 (Figure 8, lane 3), indicating that Abl kinase and PDGFR are required for phosphorylation. Simultaneous inhibition of both Abl kinase and PDGFR (Figure 8, lane 6) did not further decrease WAVE2 and Cortactin phosphorylation. In contrast, depletion of Abl kinase or inhibition of PDGFR had a more modest effect onVav2 phosphorylation (1.5–2-fold; Figure 8, lanes 3 and 5) but appeared to have an additive effect when both pathways were simultaneously affected (Figure 8, lane 6). Together, these results indicate that in the context of *C. trachomatis* infection, activation of either the Abl kinase or the PDGFR pathway leads to activation and increased tyrosine phosphorylation of WAVE2, Vav2, and Cortactin. Consistent with these results, we observed that in cells treated with STI571, phosphorylation of WAVE2, Vav2, and Cortactin was significantly diminished (Figure S5) and recruitment of phospho-Vav2 and phospho-Cortactin to EBs was impaired (data not shown). Tyrosine phosphorylation of WAVE2, Vav2, and Cortactin was not entirely abolished when Abl and PDGFR were simultaneously inhibited, suggesting that there are also Abl- and PDGFR-independent pathways of tyrosine phosphorylation.

### TARP is phosphorylated by Abl kinases

**Figure 9:**
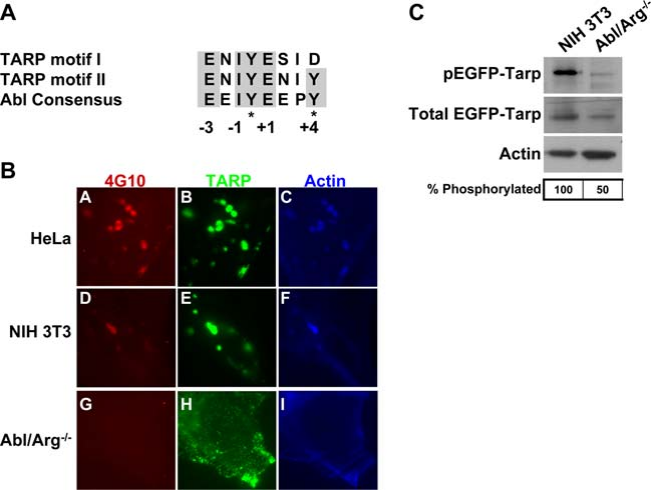
TARP is a substrate for Abl family kinases. (A) The putative TARP consensus tyrosine phosphorylation sites are compared to the Abl kinase consensus target sequence. *C. trachomatis* TARP contains six copies of a ∼50 amino acid repeat. Each repeat region encodes either motif I or motif II with 1 or 2 potential tyrosine phosphorylation sites (indicated with an asterisk). Highlighted residues indicate the high homology of the TARP motifs to consensus Abl kinase target substrates at positions −3, −1, +1, and +4. (B) HeLa, NIH 3T3, and Abl/Arg^−/−^ cells were transfected with EGFP-fused to TARP and incubated for 24 hours (green; panels B, E, H). Cells were then fixed and tyrosine phosphorylated TARP was stained with 4G10 (red; panels A, D, G) and actin was stained with phalloidin (blue; panels C, F, I). The exposure time for each filter of all images was identical. Tyrosine phosphorylation of TARP is significantly decreased in Abl/Arg^−/−^ cells. (C) Immunoblots of protein extracts from NIH 3T3 and Abl/Arg^−/−^ cells transfected with EGFP-TARP were probed with antibodies to 4G10, GFP, or actin (loading control). The fraction of phosphorylated TARP compared to total TARP was quantified by densitometry analysis for both cell types. Immunoblots shown are representative of three independent experiments. There was a significant reduction in the total TARP phosphorylated in the Abl/Arg^−/−^ cells compared to the 3T3 cells, **p<0.01 (ANOVA).

TARP is a bacterial-encoded actin nucleator likely secreted by the *Chlamydia* type III secretion system upon binding and is tyrosine phosphorylated by an as yet unidentified host kinase [19]. *C. trachomatis* L2 TARP possesses six direct repeats of approximately 50 amino acids each, and the majority of tyrosine residues are found within this region [17]. Analysis of the repeat region revealed the presence of 2 potential consensus Abl kinase target sequence motifs (E N I Y E S I D and E N I Y E N I Y) [59] (Figure 9A). To test whether TARP is a target for Abl kinase, we analyzed the phosphorylation and localization of transiently transfected enhanced green fluorescent protein fused to TARP (EGFP-TARP) in 3T3 and Abl/Arg^−/−^ cells by IF microscopy. Consistent with previous reports [16], we observed that transfected EGFP-TARP forms aggregates within the cytoplasm, is tyrosine phosphorylated, and recruits actin (Figure 9B). A qualitative reduction in the amount of tyrosine phosphorylation of EGFP-TARP in the Abl/Arg^−/−^ cells compared to the parental cells was noted (Figure 9B). In addition, TARP appeared more dispersed with fewer aggregates and localized to the cell periphery in Abl/Arg^−/−^ cells.

To rule out the possibility that the apparent decrease in TARP phosphorylation was an artifact of its more diffuse staining or varying levels of expression, parental or Abl/Arg^−/−^ cells ectopically expressing EGFP-TARP were immunoblotted with 4G10 for a quantitative analysis of phosphorylation. We controlled for differences in transfection efficiency by determining the ratio of phosphorylated EGFP-TARP to total EGFP-TARP by densitometry analysis in each cell type. There was a significant reduction in EGFP-TARP phosphorylation in the Abl/Arg^−/−^ cells compared to control cells (Figure 9C; **p<0.01 (ANOVA)). Further support of a role for Abl kinase in TARP phosphorylation was evident by the co-localization of EGFP-TARP and endogenous Abl kinase (Figure S6).

Despite a change in phosphorylation and localization for EGFP-TARP in the absence of Abl family kinases, actin was still recruited to EGFP-TARP regardless of cell type (Figure 9B). Since bacteria enter Abl/Arg^−/−^ cells as efficiently as wild type, this finding suggests that either Abl-mediated TARP phosphorylation is dispensable for entry or that the residual Abl-independent phosphorylation observed is sufficient for actin recruitment and bacterial entry.

## Discussion

Understanding the mechanisms that *Chlamydia* species use to gain entry into host cells is complicated by the ability of this obligate intracellular pathogen to enter host cells via multiple routes and by the inability to carry out classical bacterial genetics. In this study, we have circumvented these obstacles by using RNAi to carry out a large scale forward genetic screen in *D. melanogaster*, a surrogate host with less functional redundancy, to identify host proteins required for early steps in *C. trachomatis* infection. Our screen confirmed some previously known host targets and has, most importantly, identified for the first time the activation of PDGFR and Abl kinase signaling pathways as key events in the pathogenesis of *C. trachomatis* infections. We demonstrate that in mammalian cells, PDGFRβ functions as a receptor for *C. trachomatis* binding and that once bound, bacterial internalization can occur either through activation of PDGFRβ or through independent activation of Abl kinase. Activation of these kinases culminates in phosphorylation of the Rac guanine nucleotide exchange factor, Vav2, and several actin nucleators, including WAVE2 and Cortactin, that ultimately promote efficient uptake of this obligate intracellular parasite.

The initial step in *Chlamydia* binding is thought to be a reversible, electrostatic interaction with heparan sulfate-like glycosaminoglycans followed by an irreversible interaction with an unknown receptor [60]. We provide compelling data that at least one receptor for *C. trachomatis* binding is PDGFRβ. We demonstrate that RNAi-mediated depletion of PDGFRβ or addition of a neutralizing antibody to PDGFRβ significantly decreases bacterial binding to mammalian cells. Consistent with its role as a receptor, phosphorylated PDGFRβ is recruited to the site of EB binding.

There are several possible models to explain the interactions between *C. trachomatis* and PDGFRβ. The bacterium may bind directly to PDGFRβ. Alternatively, the interactions may be indirect. For example, EBs could bind directly to the growth factor receptor ligand (PDGF), which would in turn facilitate binding and activation of PDGFRβ. Another possible scenario is that heparan sulfate (either on the surface of *C. trachomatis* or on the surface of host cells) could bind to PDGF, facilitating subsequent interaction with PDGFRβ. Finally, EBs, heparan sulfate, PDGF, and PDGFRβ could form a complex, with heparan sulfate and PDGF serving as bridging molecules. Although EGFR was not involved in *C. trachomatis* binding to host cells under our experimental conditions, suggesting specificity for PDGFRβ we cannot rule out a possible role for other growth factor receptor interactions.

The biological properties of PDGFRβ fit well with the known characteristics of EB binding and entry. The receptors are ubiquitously expressed in cultured cells, consistent with the known ability of *C. trachomatis* to enter most cell types *in vitro*. PDGFRβ is known to be highly expressed in the uterus and ovaries as well as in macrophages, tissues and cells susceptible to *C. trachomatis* infections *in vivo* [61]. PDGFRβ internalization occurs by both clathrin-dependent and clathrin-independent pathways (reviewed in [62]), consistent with the reported diversity in *C. trachomatis* internalization mechanisms (reviewed in [60]). PDGFRβ signals to regulators of the actin cytoskeleton [22]–[26] that are activated in response to *C. trachomatis* infection, including Vav2 (this work), Cortactin (this work), WAVE2 (this work and [56]), and Rac [56],[63]. PDGF can bind heparan sulfate proteoglycans, and this interaction can enhance PDGF-induced signaling of PDGFR [64],[65], potentially explaining the contribution of this heparan sulfate to *Chlamydia* binding. Finally, activation of and entry through a growth factor receptor pathway may serve to promote host cell survival and prevent apoptosis early during infection, which is vital for the successful growth and dissemination of an obligate intracellular parasite. Indeed, the phosphatidylinositol-3 kinase (PI3K) pathway contributes to resistance of *C. trachomatis* infected cells to apoptosis [66], although the mechanism by which this pathway is activated remains to be determined. Since PDGFR can exert an antiapoptotic effect in a PI3K-dependent manner [67], we speculate that bacterial binding to PDGFRβ may activate the PI3K pathway.

The results presented here indicate that *C. trachomatis* binding leads to activation of PDGFRβ and Abl kinase signaling pathways, which operate in a redundant manner to ensure failsafe and efficient uptake of this obligate intracellular parasite into mammalian cells. While inhibition of either Abl kinase or PDGFR alone has a minimal effect on bacterial entry, inhibition of both kinases either by STI571 treatment or by inhibiting PDGFRβ in cells where Abl is deleted or depleted significantly decreases internalization. Furthermore, a STI571-resistant allele of Abl kinase is capable of supporting entry in the presence of drug, indicating that Abl kinase alone is sufficient for entry. We note that in S2 cells, depletion of Abl kinase alone is sufficient to decrease vacuole formation. This may reflect the absence of functional redundancy in *Drosophila* or may indicate an additional essential role for Abl kinase in post-entry events.

Our results indicate that during infection, *C. trachomatis*-induced activation of PDGFRβ is not necessary for activation of Abl kinase even though PDGFRβ signaling has been shown to activate Abl kinase in other settings [44],[68],[69]. How Abl kinase is activated upon EB binding remains to be determined. Abl kinase activation may occur through activation of another as yet unidentified surface receptor or it may be activated by TARP or other translocated bacterial effectors [70],[71].

Our work demonstrates that *C. trachomatis* infection leads to tyrosine phosphorylation and recruitment of several key molecules involved in Rac-dependent actin rearrangements that are known to be regulated by PDGFR and Abl kinase [20]–[26], [72]–[75]. These include Vav2, a Rac GEF as well as WAVE2 and Cortactin, activators of the Arp2/3 complex in the Rac pathway. Although our IF data using the 4G10 antibody (Figures 5 and 6) suggests that Abl kinase is the major kinase responsible for phosphorylating EB-associated proteins, our western blot data (Figure 8) indicates that PDGFR activity also contributes to tyrosine phosphorylation of WAVE2, Vav2, and Cortactin. Since Abl kinase and PDGFR function redundantly for *C. trachomatis* entry, the indispensable role of Abl kinase in tyrosine phosphorylation of EB-associated proteins and the dispensable role of Abl in *C. trachomatis* invasion can be explained by: (i) Abl kinase may phosphorylate a large fraction of EB-associated proteins, while PDGFR phosphorylates a subset of functionally redundant proteins, (ii) some of the proteins phosphorylated by Abl kinase may have multiple tyrosine residues (such as TARP), making Abl appear to be the major kinase, and/or (iii) not all Abl targets play a role in entry.

To our knowledge, Vav2 has not been previously implicated in *C. trachomatis* infection. We have previously observed co-localization of Cortactin with inclusions [15]. While this manuscript was in preparation, Hybiske *et al* reported that depletion of Cortactin results in a modest decrease in entry [76], and Carrabeo *et al* demonstrated that WAVE2 and Arp3 colocalize with EBs and are required for entry [56]. We now link these signaling cascades involving Rac and Arp2/3 activators to upstream events that include EB binding to and activation of PDGFR as well as activation of Abl kinase. We conclude that activation of Abl kinase and PDGFR are necessary to ensure efficient recruitment and activation of downstream signaling molecules, including WAVE2, Vav2, and Cortactin, which mediate actin polymerization and entry.

The exact relationship between Abl activation, phosphorylation of the putative type III secreted effector TARP, entry, and vacuole formation is likely to be complex. Though our findings demonstrate that Abl kinase phosphorylates TARP, inhibition of Abl kinase by several different methods did not prevent entry. One explanation for this result is, as suggested by others [16],[19], that TARP phosphorylation is not required for entry; instead, it could be important for a post entry function, such as inclusion trafficking and/or fusion. This observation could explain why the significantly diminished phosphorylation of EBs in Abl/Arg^−/−^ cells did not affect entry, especially given that TARP encodes many tyrosine residues that could serve as putative phosphorylation sites. Alternatively, TARP phosphorylation was not completely abolished in the Abl/Arg^−/−^ cells, suggesting that other tyrosine kinases, such as Src family kinases, may target TARP. This residual TARP phosphorylation could be sufficient to mediate bacterial entry. Interestingly, Src family kinases can be activated by PDGFR signaling and regulate cytoskeletal dynamics (reviewed in [77]), thus the PDGFR-Src pathway could function redundantly with Abl kinase to promote entry.

We speculate that Abl-dependent phosphorylation of TARP may provide docking sites for recruitment of additional SH2 containing signaling molecules that may increase the efficiency of entry. This could include setting up a positive feedback pathway similar to what has been recently reported for Tir phosphorylation during pedestal formation by Enteropathogenic *Escherichia coli* [78]. Initial phosphorylation of TARP would lead to recruitment of additional kinase molecules as well as new TARP molecules, culminating in the recruitment of actin and actin polymerizing factors [78]. Alternatively, by virtue of its four polyproline motifs (PxxxP) [19], TARP could bind to the SH3 domains of both Vav2 and Abl kinase. This interaction would serve to bring Abl kinase in contact with Vav2 and ensure efficient Rac activation, a mechanism that has been proposed for Vav activation by the Murine gamma-herpesvirus 68 latency protein M2 [79]. It is also possible that key role of Abl kinase during entry is to phosphorylate and/or recruit other actin polymerization mediators (i.e. WAVE2, Vav2, and Cortactin).

The modulation of signaling pathways involving Abl kinase and bacterial internalization is an emerging theme among important human pathogens, including *Shigella flexneri* [80] and Group B coxsackievirus [81]. In contrast to these pathogens where Abl kinase is the only kinase required for entry, our findings demonstrate that entry can occur either via an Abl kinase-dependent pathway or through activation of PDGFRβ. Our results further suggest that these two pathways function in parallel and are thus functionally redundant. Since STI571 can inhibit both Abl and PDGFR kinases, this finding may explain why bacterial entry is diminished with this drug but unaffected in Abl/Arg^−/−^ cells. Although we cannot rule out the possibility that other STI571-inhibitable kinases play a role in *C. trachomatis* entry, such as c-Fms and Lck kinase [82],[83], these targets are not, to the best of our knowledge, expressed in HeLa or NIH 3T3 cells, [84]–[87]). Furthermore, the fact that we can recapitulate the entry defect observed with STI571 by depleting Abl kinase and inhibiting PDGFR in the same cell using alternative means (ie. Treatment of Abl/Arg^−/−^ with AG1295, a specific PDGFR inhibitor) provides compelling evidence that these proteins are likely the main kinases affected by STI571 in the context of *C. trachomatis* entry.

**Figure 10:**
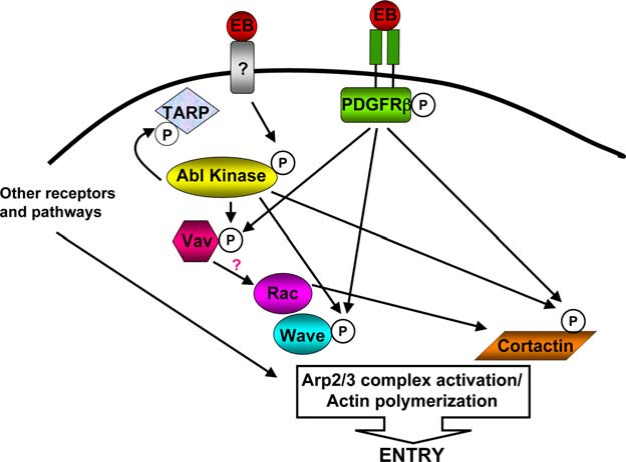
Model for *C. trachomatis* binding and internalization. EBs bind to and activate PDGFRβ, possibly via heparan sulfate and/or PDGF. Abl kinase is recruited to and activated at the site of EB binding in a PDGFRβ-independent manner and phosphorylates TARP. The combined activation of PDGFRβ and Abl kinase leads to phosphorylation and activation of Vav2, WAVE2, and Cortactin. Rac and Arp 2/3 are recruited to the site of entry, possibly through interaction with TARP or via PDGFRβ and/or Abl kinase signaling. Actin polymerization is stimulated through the WAVE2/Arp2/3 pathway, Cortactin/Arp2/3 pathway, and/or directly by TARP. Other receptors and pathways converge and/or synergize with these molecules to ensure efficient Rac and Arp2/3 activation for *C. trachomatis* uptake.

Our results are consistent with the following model for *C. trachomatis* entry into host cells (Figure 10). *C. trachomatis* L2 binds to and activates PDGFRβ, possibly via heparan sulfate and/or PDGF. Abl kinase is recruited to and activated at the site of EB binding in a PDGFR-independent manner and phosphorylates TARP. Activation of PDGFR and Abl kinase leads to recruitment and activation of downstream targets, including Vav2, WAVE2, and Cortactin. Rac and Arp 2/3 are recruited to the site of entry. Actin polymerization is stimulated through the WAVE2/Arp2/3 pathway, Cortactin/Arp2/3 pathway, and/or directly by TARP. Since binding and entry of *C. trachomatis* was not completely abolished under our experimental system, this bacterium most likely utilizes other receptors and pathways that converge with these molecules to ensure efficient Rac and Arp2/3 activation for *Chlamydia* uptake. In addition, Abl-dependent TARP phosphorylation may contribute to critical events after entry that are required for this obligate intracellular parasite to survive and replicate in the host cell.

In summary, we have applied a genome-wide RNAi-based forward genetic screen to discover that *C. trachomatis* hijacks PDGFRβ and Abl kinase to modulate the host cytoskeleton in order to efficiently to enter host cells.

## Materials and Methods

### Reagents

Cholesterol, Methyl-beta-cyclodextrin (MβCD), heparin, and Concanavalin A (ConA) were purchased from Sigma-Aldrich. Recombinant human PDGF-BB and EGF were purchased from R&D Systems. AG1295 was obtained from Calbiochem. The concentration for AG1295 and STI571 was 40 µM, unless otherwise indicated, as these doses provided maximal inhibition without affecting cell viability. GFP-TARP construct was a kind gift from R. Carabeo and has been previously described [16]. The pIRESSKII EGFP (Clontech), with bases 1870–1910 removed, has been described previously [88]. HA-tagged Abl, untagged wild type Abl, and untagged Abl STI571R (also known as Abl-T351) have been previously described [55]. Antibodies were obtained from the following sources: mouse anti-*Chlamydia* FITC conjugate (Meridian Diagnostics), goat anti-*C. trachomatis* MOMP (Cortex Biochem), rabbit anti-*Chlamydia* LPS (Cortex Biochem), mouse anti-GAPDH (Chemicon), goat anti-actin (Santa Cruz), rabbit anti-PDGFRβ (Santa Cruz), goat anti-PDGFRβ (R&D), rabbit anti-phospho-PDGFRβ (Tyr751) (Cell Signaling), rabbit anti-EGFR (Santa Cruz), mouse anti-Abl (Santa Cruz), rabbit anti-Abl-pY412 (Cell Signaling Technology), mouse anti-4G10 (Upstate), mouse anti-Cortactin (Upstate), rabbit anti-Vav2 (Santa Cruz), goat anti-WAVE2 (Santa Cruz), mouse anti-gfp (Roche), mouse and rabbit anti-HA (Roche), mouse anti-gfp (Roche), mouse anti-Crk (BD Transduction), rabbit anti-CrkII (Tyr221) (Cell Signaling), rabbit anti-goat IgG HRP (Calbiochem), goat anti-rabbit IgG HRP (Amersham Biosciences), goat anti-mouse IgG HRP (Amersham Biosciences), donkey anti goat Alexa 594 (Molecular Probes), chicken anti-mouse 594 (Molecular Probes), donkey anti-goat Alexa 488 (Molecular Probes), and donkey anti-rabbit Alexa 488 (Molecular Probes). Texas red- or Alexa 350-conjugated phalloidin were obtained from Molecular Probes. All siRNA's were obtained from Santa Cruz Biotechnology, Inc: Abl kinase (sc-29843), PDGFRβ (sc-29442), EGFR (sc-29301), and Control siRNA-A (sc37007).

### Cell culture and *C. trachomatis* propagation

HeLa 229 cells and L929 cells were obtained from ATCC and passaged as previously described [89]. NIH 3T3 cells were obtained from ATCC. 3T3 cells derived from Abl/Arg^−/−^ mice [49] were maintained at 37°C with 5% CO_2_ in DMEM containing 20% Fetal Bovine Serum (FBS). *Drosophila* S2 cells were kind gifts of Ron Vale (UCSF) and cultured in Schneider's medium supplemented with 10% FBS. *C. trachomatis* serovar LGV L2 was propagated in L929 cells. *C. trachomatis* EBs were harvested from infected cells and purified using a renografin step-gradient essentially as described [90].

### RNAi screen

The dsRNA library used in this screen has been described previously [28]. S2 cells were placed into 96-well plates at a density of 40,000 cells per well in a culture volume of 150 µl per well. dsRNA was added to a final concentration of 10 µg/ml, and the cells were incubated for four days at 28°C to allow for depletion of the corresponding gene product. For primary and secondary screens, 1×10^5^ dsRNA-treated S2 cells were replated in 96-well plastic plates. S2 cells were infected with *C. trachomatis* L2 (MOI of ∼100) in the presence of 50 µM MβCD-cholesterol and incubated at 28°C. After 1 h of infection, bacteria were removed, cells were rinsed with Phosphate Buffered Saline (PBS), fresh media supplemented with 1 mg/ml heparin was added, and cells were incubated for 48 hours. The *Chlamydia*-infected S2 cells were replated onto ConA-coated glass bottom 96 well dishes and allowed to adhere for 30 min, washed with PBS, fixed in ice-cold Methanol for 5 min, stained with anti-*Chlamydia* antibody conjugated to FITC for 1h, and counterstained with Evan's blue to visualize cells. Infected cells were visually screened for an apparent increase or decrease in vacuole formation, and changes in cell number were also noted. For secondary screens, two investigators screened wells independently and discrepancies were resolved with further analysis.

### Immunofluorescence studies

HeLa, NIH 3T3, and Abl/Arg^−/−^ cells were grown on glass coverslips in 24 well plates and infected with *C. trachomatis* as described in the text. Cells were fixed in 4% paraformaldehyde (PFA) and permeabilized with 0.1% Triton containing 0.05% sodium dodecyl sulfate-polyacrylamide (SDS). After blocking in 1% bovine serum albumin/PBS, cells were incubated with the appropriate antibody for 1 hour, washed three times, and incubated for 2 hours with the appropriate fluorophore-conjugated secondary. Coverslips were mounted in Vectashield mounting media containing DAPI (Vector Laboratories) to identify bacteria and host cell nuclei. Images for all immunofluorescent studies were acquired at a magnification of ×1,000 under oil immersion or ×400 with a Nikon Eclipse TE2000-E fluorescence microscope, using a CCD camera and processed by Simple PCI imaging software (Compix, Inc.). For each set of experiments, the exposure times were identical for all images. Images were processed with Adobe Photoshop CS.

### Immunoprecipitation

Cells were lysed in lysis buffer (50 mM Tris HCl, pH 7.5, 150 mM NaCl, 0.1% SDS, 1% Nonidet P-40, 1% sodium deoxycholate, 1mM sodium orthovanadate, 1 mM sodium fluoride, 1 mM okadaic acid, and Complete protease inhibitors: Roche Diagnostics). After centrifugation at 20, 800 *g* for 5 minutes to remove cell debris, the supernatants were transferred to fresh tubes. Supernatants containing the indicated antibody preconjugated to Protein G Sepharose TM 4 Fast Flow (GE HealthCare) were incubated for 1 h at 4°C with gentle rocking. Immunoprecipitates were recovered by centrifugation, washed 3 times in lysis buffer, eluted by boiling in SDS sample buffer, and immunoblotted.

### Immunoblot analysis

Proteins were separated on 8% or 10% sodium dodecyl sulfate-polyacrylamide gel electrophoresis (SDS-PAGE) gels and transferred to 0.45 µm Trans-blot nitrocellulose membranes (BioRad Laboratories). Membranes were blocked with 3% milk (Upstate) and probed with the indicated antibodies. Proteins were detected by ECL (Amersham Biosciences) according to the manufacturer's protocol. For quantitation, bands were analyzed using ImageQuant software (Molecular Dynamics, Foster City, CA).

### RNAi in mammalian cells

HeLa cells grown in 6 well plates were transfected with the indicated siRNA (Santa Cruz) according to manufacturer's protocol and incubated for 24 hours. For phosphorylation assays, siRNA-treated cells were trypsinized following the 24 hour incubation, and replated into 24 well plates containing glass coverslips. At 40 hours post transfection, cells were infected with *Chlamydia* as described above and either analyzed for EB-associated tyrosine phosphorylation by 4G10 antibody staining, assessed for internalization efficiency as described above, or subjected to immunoblot analysis or immunoprecipitation with the indicated antibody. In some cases, cells were pretreated with STI571 or AG1295 for 1 hour prior to and during infection. A portion of the siRNA-treated cells was immunoblotted to determine efficiency of protein depletion.

### Phosphorylation assay

HeLa, NIH 3T3, and Abl/Arg^−/−^ cells were grown on glass coverslips in 24 well plates and infected with *C. trachomatis.* For immunofluorescence analysis of EB-associated tyrosine phosphoproteins, cells were fixed in 4% paraformaldehyde (PFA) and permeabilized with 0.1% Triton containing 0.05% SDS. After blocking in 1% bovine serum albumin/phosphate-buffered saline (PBS), cells were incubated with 4G10 antibody for 1 hour. Cells were washed three times and then incubated for 2 hours with the fluorophore-conjugated anti-mouse secondary and in some cases, fluorophore-conjugated phalloidin. Coverslips were mounted in Vectashield mounting media containing DAPI (Vector Laboratories) to identify bacteria. For quantitation of EB-associated phosphorylation, images were obtained from either Texas red and DAPI or FITC and DAPI channels, depending on the fluorophore used. Merged channels were analyzed. Total EBs from the DAPI channel were enumerated and the efficiency of phosphorylation was calculated using the formula (Single channel (red or green)/total EBs)×100. A minimum of 5 fields containing an average of 9 cells was analyzed per treatment. The date was compiled from 3 experiments and is presented as means±standard error.

### Inside out staining

Inside out staining was performed as described in [11],[91] with modifications. Cells were grown overnight on glass coverslips in 24 well plates, drug-treated as indicated in the text or siRNA-treated as described above, and subsequently infected with *Chlamydia* at an MOI = 10 for 1 hour at 37°C to allow for attachment and internalization. For antibody inhibition assays, cells were preincubated with the indicated neutralizing antibody or the isotype-matched control antiserum for 1 hour prior to addition of bacteria. Infected cells were washed in PBS and fixed in 1% PFA, gentle conditions that prevent permeabilization of the host cell. After fixation, cells were blocked in 2% FBS/1% Fish Skin Gelatin (FSG)/PBS for 1 hour and then incubated with goat anti-MOMP antibody for 1 hour followed by incubation with donkey anti-goat Alexa 488 antibody to stain external EBs. Cells were then permeabilized with 0.1% Triton X-100, blocked again, and incubated with rabbit anti-*Chlamydia* LPS antibody followed by incubation with goat anti-rabbit Alexa 594 antibody to stain intracellular and extracellular EBs. The host cell was visualized by staining the actin cytoskeleton with phalloidin-Alexa 350 to visualize the cell. The coverslips were then mounted and visualized by immunofluorescence microscopy as described above. All internalization and binding assay images were acquired with a Nikon TE-2000E microscope using a 100× objective and saved in RGB 24-bit tiff format. Images were collected form Texas-red, FITC and DAPI channels and merged. Merged images were imported into Adobe Photoshop V.8 where the Pencil tool was used to mask all bacteria that were not cell-associated. Enhanced images were analyzed using MetaMorph (Molecular Devices, Sunnyvale, CA). In MetaMorph, merged images were first separated using an RGB Color Separator into three 8-bit monochrome tiff images (Texas Red, FITC and DAPI). The Image Multiplication function was then used to create two 16-bit tiff images from the Texas Red and FITC channels (one Texas Red-Texas Red multiplication and one Texas Red-FITC multiplication). The 16-bit images were analyzed with the Count Nuclei function and a grey level setting above background ranging from 12,000 to 18,000, depending on the level of contrast and signal strength. The Texas Red-Texas Red multiplication was scored as total cell-associated EBs, whereas the Texas Red-FITC multiplication was scored as extracellular EBs. The efficiency of internalization was calculated using the formula ((Texas Red-Texas Red−Texas Red-FITC)/(Texas Red-Texas Red))×100. A minimum of 6 fields containing an average of 10 cells were analyzed per treatment. The data were compiled from at least 3 independent experiments and were normalized to no drug treatment samples and are presented as means±standard error.

### Transfection studies

HeLa, NIH 3T3, or Abl/Arg^−/−^ cells were seeded on 12 mm glass coverslips in 6 well plates and transfected with the indicated plasmid constructs using Effectene (Invitrogen) following manufacturer's instructions. Expression from the EGFP and EGFP-TARP constructs were allowed to proceed for 24 hours at 37°C as previously described [16] and expression from the all Abl kinase constructs was allowed to proceed for 48 hours as previously described [55] before infecting with *C. trachomatis*. Coverslips from transfected cells were processed for immunofluorescence as described above. Actin staining was performed using fluorophore-conjugated phalloidin (Molecular Probes) for 2 hours. The remaining cells were harvested and immunoblotted as described above.

### Statistical analysis

Data represented the mean±standard error of *n* experiments. Statistical analysis was performed using the software program Instat. The significance between groups was determined by ANOVA. A *p* value less than 0.05 was considered to be statistically significant.

### Accession numbers

The FlyBase (http://flybase.bio.indiana.edu/search/) accession numbers for the genes and gene products discussed in this paper are Pvr (FBgn0032006), Abl kinase (FBgn0000017), SCAR/WAVE (FBgn 0041781), Vav (FBgn 0040068), and Cortactin (FBgn 0025865). The NCBI Entrez (http://www.ncbi.nlm.nih.gov/gquery/gquery.fcgi) accession numbers for human genes discussed in this paper are PDGFRβ (NP_002600), PDGF-B (NP_002599, NP_148937), Abl kinase (NP_005148, NP_009297), WAVE2 (NP_008921), Vav2 (NP_003362), Cortactin (NP_612632, NP_005222), EGFR (NP_958441, NP_958439), and TARP (AY623902).

## Supporting Information

Table S1 Genes identified in RNAi screen.
Genes were organized according to phenotypes as observed by immunofluorescence microscopy. Gene function was as described in Flybase, information hyperlinked over proteins (iHOP), or by gene homologies. Human gene homology was determined by iHOP.
(0.04 MB XLS)

Figure S1 PDGFR is not necessary for activation of Abl kinase upon *C. trachomatis* infection or for tyrosine phosphorylation of proteins associated with EBs.
HeLa cells were infected with *C. trachomatis* for 1 hr in the absence or presence of STI571 or AG1295.
(A) The percentage of EBs associated with phospho-Abl were quantified by IF using the phospho-Abl Y412 antibody, and values are shown as the mean±s.e.m. Data are from two independent experiments, and approximately 500 EBs were counted. ***p<0.001 compared with DMSO-treated and AG1295-treated HeLa cells (ANOVA).
(B) The percentage of EBs associated with tyrosine phosphorylated proteins was quantified by IF using the 4G10 antibody, and values are shown as the mean±s.e.m. Data are from two independent experiments, and approximately 500 EBs were counted. ***p<0.001 compared with DMSO-treated and AG1295-treated HeLa cells (ANOVA).
(1.65 MB TIF)

Figure S2 Abl Kinase is necessary for tyrosine phosphorylation of proteins associated with EBs.
The percentage of EBs associated with tyrosine phosphorylated proteins was quantified by IF using the 4G10 antibody in *C. trachomatis*-infected (A) NIH 3T3 and Abl/Arg^−/−^ or (B) control and Abl siRNA-treated HeLa cells treated with DMSO or STI571. Values are shown as the mean±s.e.m. Data are from at least three independent experiments, and approximately 1000 EBs were counted. ***p<0.001 (ANOVA) compared to DMSO-treated NIH 3T3 (A) or DMSO-treated control siRNA (B).
(1.79 MB TIF)

Figure S3 Abl Kinase is sufficient for tyrosine phosphorylation of proteins associated with EBs.
NIH 3T3 (panels A–H) and Abl/Arg^−/−^ cells (panels I–P) were transfected with plasmids encoding HA-Abl (panels E–H and M–P) or EGFP (panels A–D and I–L) for 48 hours, infected with *C. trachomatis* for 1 hour, and then stained for tyrosine phosphorylation using 4G10 (panels A, E, I, and M; red in merge). Cells expressing HA-Abl were visualized by staining with an anti-HA antibody (panels F and N; green in merge). Bacteria and host DNA were detected using DAPI (panels C, G, K, and O; blue in merge). The exposure time for each filter of all images was identical. Expression of Abl kinase is sufficient to restore EB-associated tyrosine phosphorylation in Abl/Arg^−/−^ cells.
(8.40 MB TIF)

Figure S4 Dose-dependent inhibition of Abl kinase activity by STI571 treatment.
HeLa cells were pretreated with DMSO or the indicated concentration of STI571 for 1 hr and subsequently infected with *C. trachomatis* in the presence of DMSO or STI571. Abl kinase activity was assessed by analyzing the phosphorylation of CrkII, an Abl kinase substrate. CrkII was immunoprecipitated from lysates and immunoblotted with anti-phospho-CrkII (Tyr221) antibody to assess phosphorylation. Blots were reprobed with total CrkII antibody to determine total protein amounts. All samples were run on the same gel and exposed the same amount of time. The percentage of phosphorylated protein compared to total protein was quantified by densitometry analysis and normalized relative to *C. trachomatis*-infected samples. Immunoblots shown are representative of three independent experiments. Abl kinase activity is inhibited with increasing doses of STI571.
(2.79 MB TIF)

Figure S5 *C. trachomatis* -induced phosphorylation of WAVE2, Vav2, and Cortactin is diminished by STI571.
HeLa cells were treated with DMSO or STI571 for 1 hour, and then subsequently infected with *C. trachomatis* for 1 hour. WAVE2 and Cortactin were immunoprecipitated from lysates and immunoblotted with 4G10 to assess phosphorylation. Blots were reprobed with the indicated antibody to determine total protein amounts. Lysates from the same set of samples were probed with an anti-pVav2 and total Vav2 antibodies. The percentage of phosphorylated protein compared to total protein was quantified by densitometry analysis and normalized relative to *C. trachomatis*-infected samples. Immunoblots shown are representative of three independent experiments. WAVE2, Vav2, and Cortactin phosphorylation is diminished by STI571.
(2.51 MB TIF)

Figure S6 Colocalization of Abl kinase and TARP.
HeLa cells were transfected with a vector encoding EGFP (A–D) or EGFP-TARP (E–H) for 24 hours. Cells were then fixed and stained with anti-Abl (B and F; red in merge). Actin was stained with phalloidin (C and G; blue in merge). The exposure time for each filter of all images was identical. Note the colocalization of EGFP-TARP and Abl kinase.
(9.24 MB TIF)
