# Correction: RNA Interference Screen Identifies Abl Kinase and PDGFR Signaling in *Chlamydia trachomatis* Entry

**DOI:** 10.1371/annotation/d041a126-3e08-40ab-bee4-3448404b07b3

**Published:** 2013-08-06

**Authors:** Cherilyn A. Elwell, Alhaji Ceesay, Jung Hwa Kim, Daniel Kalman, Joanne N. Engel

The following should be included as the first sentence of the abstract:

“The strain designated Chlamydia trachomatis serovar L2 that was used for experiments in this paper is Chlamydia muridarum, a species closely related to C. trachomatis (and formerly termed the Mouse Pneumonitis strain of C. trachomatis). This conclusion was verified by deep sequencing and by PCR using species-specific primers. All data presented in the results section that refer to C. trachomatis should be interpreted as referring to C. muridarum. Since C. muridarum TARP lacks the consensus tyrosine repeats present in C. trachomatis TARP, we cannot make any conclusions about the role of TARP phosphorylation and C. muridarum entry. However, the conclusion that C. trachomatis L2 TARP is a target of Abl kinase is still valid as these experiments were performed with C. trachomatis L2 TARP.”

