# Supplementary figures and images for: RNA Interference Screen Identifies Abl Kinase and PDGFR Signaling in Chlamydia trachomatis Entry
Source: PLoS Pathog. 2008 Mar 7;4(3):e1000021. doi: 10.1371/journal.ppat.1000021 (PMC2267011; doi:10.1371/journal.ppat.1000021)

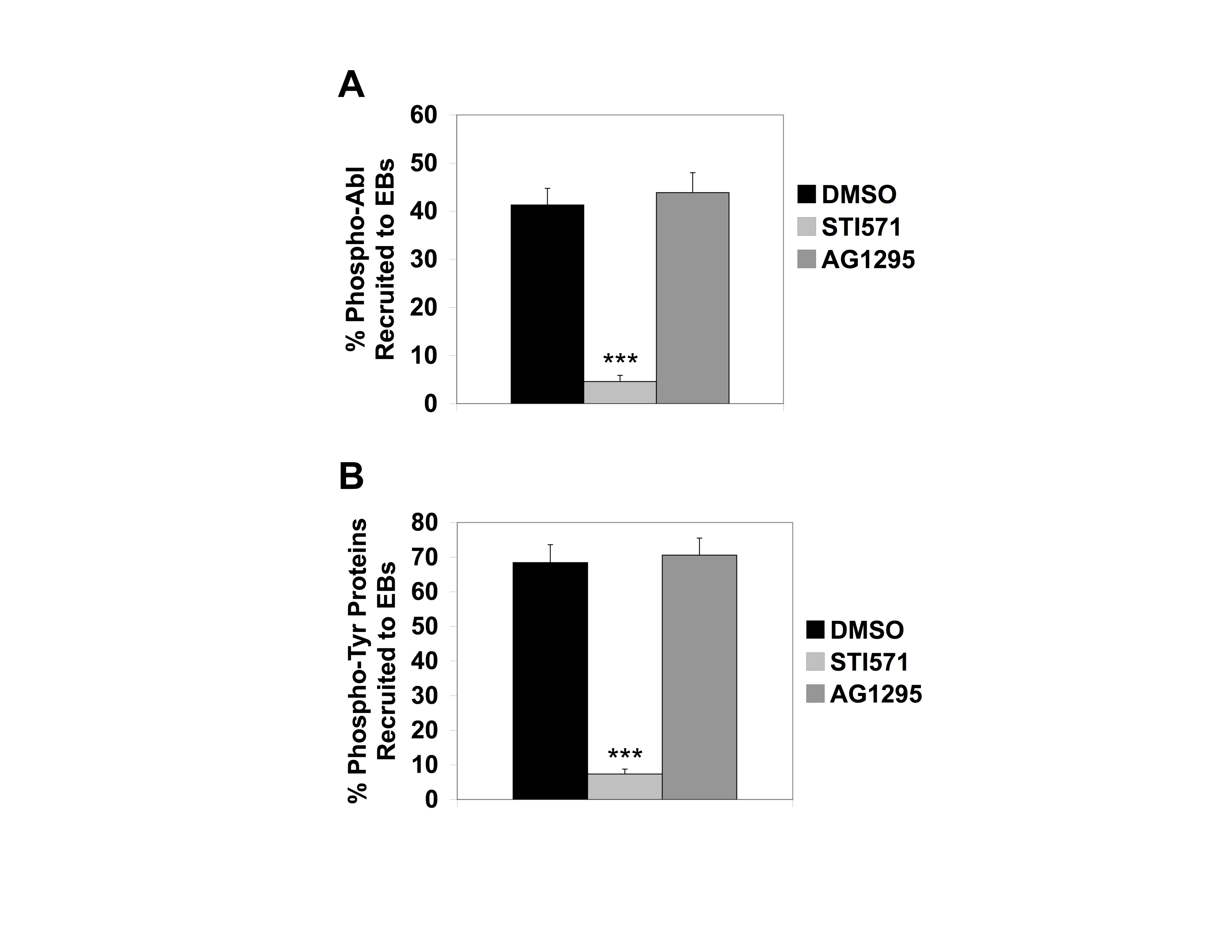

Supplement: Figure S1 — PDGFR is not necessary for activation of Abl kinase upon C. trachomatis infection or for tyrosine phosphorylation of proteins associated with EBs. HeLa cells were infected with C. trachomatis for 1 hr in the absence or presence of STI571 or AG1295. (A) The percentage of EBs associated with phospho-Abl were quantified by IF using the phospho-Abl Y412 antibody, and values are shown as the mean±s.e.m. Data are from two independent experiments, and approximately 500 EBs were counted. ***p<0.001 compared with DMSO-treated and AG1295-treated HeLa cells (ANOVA). (B) The percentage of EBs associated with tyrosine phosphorylated proteins was quantified by IF using the 4G10 antibody, and values are shown as the mean±s.e.m. Data are from two independent experiments, and approximately 500 EBs were counted. ***p<0.001 compared with DMSO-treated and AG1295-treated HeLa cells (ANOVA). (1.65 MB TIF) [file ppat.1000021.s002.tif]

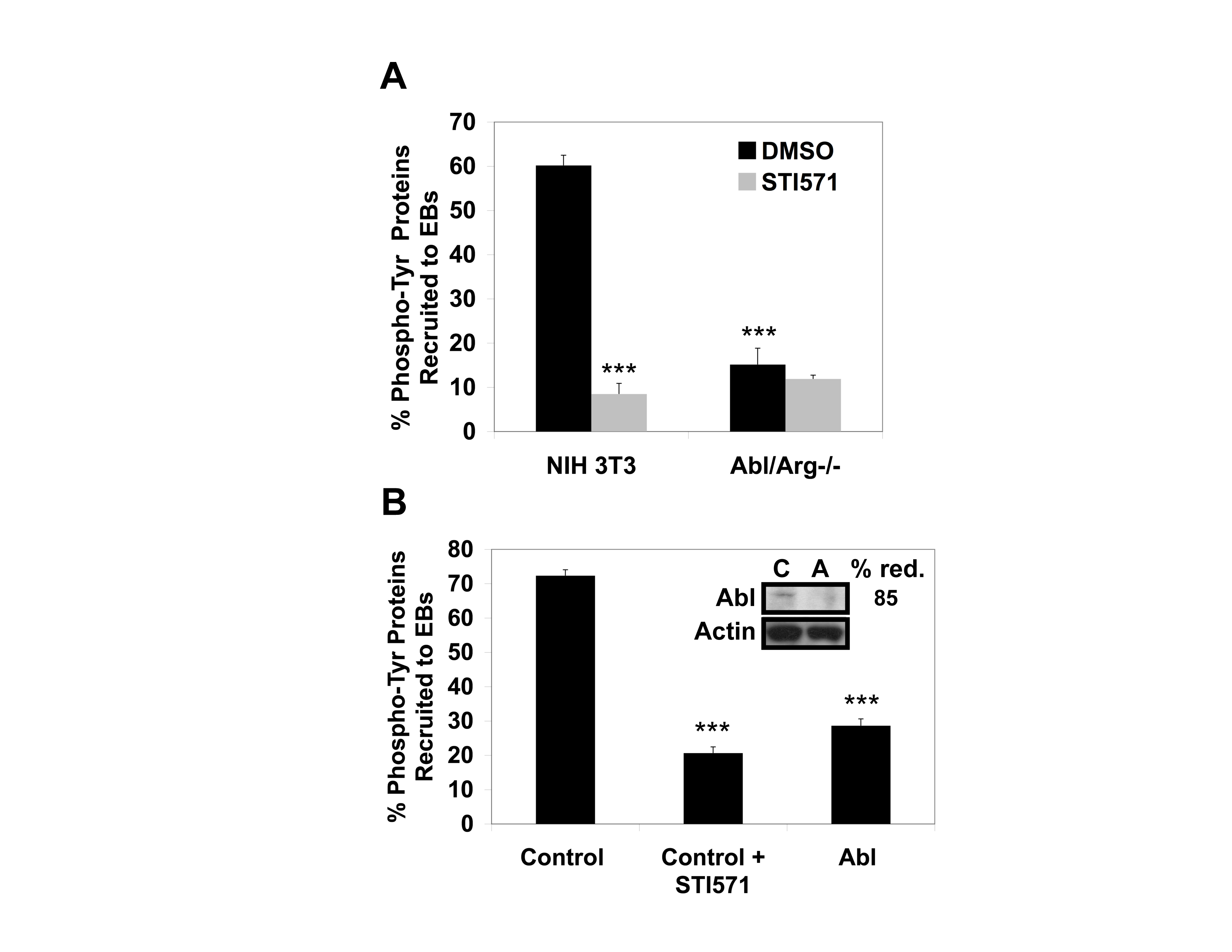

Supplement: Figure S2 — Abl Kinase is necessary for tyrosine phosphorylation of proteins associated with EBs. The percentage of EBs associated with tyrosine phosphorylated proteins was quantified by IF using the 4G10 antibody in C. trachomatis-infected (A) NIH 3T3 and Abl/Arg−/− or (B) control and Abl siRNA-treated HeLa cells treated with DMSO or STI571. Values are shown as the mean±s.e.m. Data are from at least three independent experiments, and approximately 1000 EBs were counted. ***p<0.001 (ANOVA) compared to DMSO-treated NIH 3T3 (A) or DMSO-treated control siRNA (B). (1.79 MB TIF) [file ppat.1000021.s003.tif]

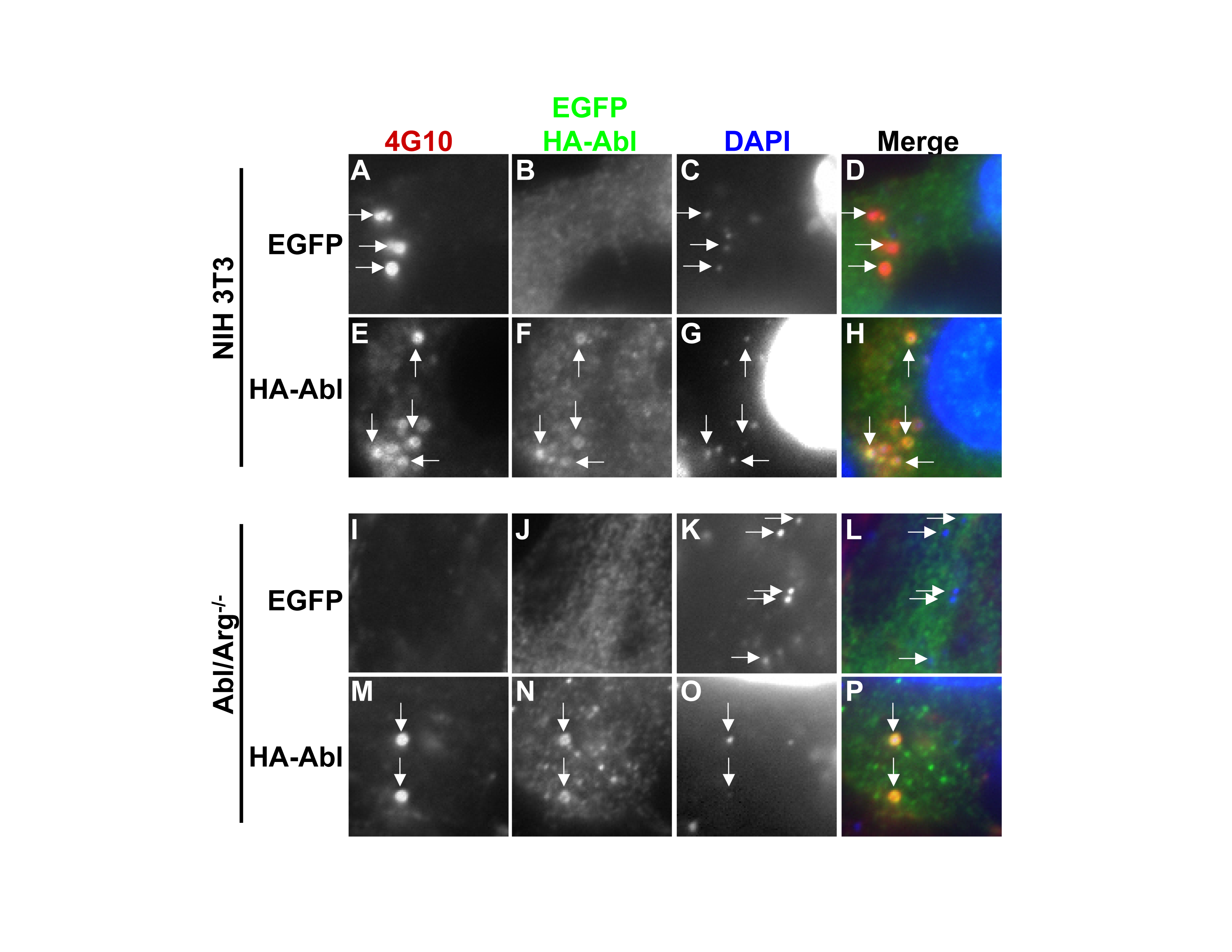

Supplement: Figure S3 — Abl Kinase is sufficient for tyrosine phosphorylation of proteins associated with EBs. NIH 3T3 (panels A–H) and Abl/Arg−/− cells (panels I–P) were transfected with plasmids encoding HA-Abl (panels E–H and M–P) or EGFP (panels A–D and I–L) for 48 hours, infected with C. trachomatis for 1 hour, and then stained for tyrosine phosphorylation using 4G10 (panels A, E, I, and M; red in merge). Cells expressing HA-Abl were visualized by staining with an anti-HA antibody (panels F and N; green in merge). Bacteria and host DNA were detected using DAPI (panels C, G, K, and O; blue in merge). The exposure time for each filter of all images was identical. Expression of Abl kinase is sufficient to restore EB-associated tyrosine phosphorylation in Abl/Arg−/− cells. (8.40 MB TIF) [file ppat.1000021.s004.tif]

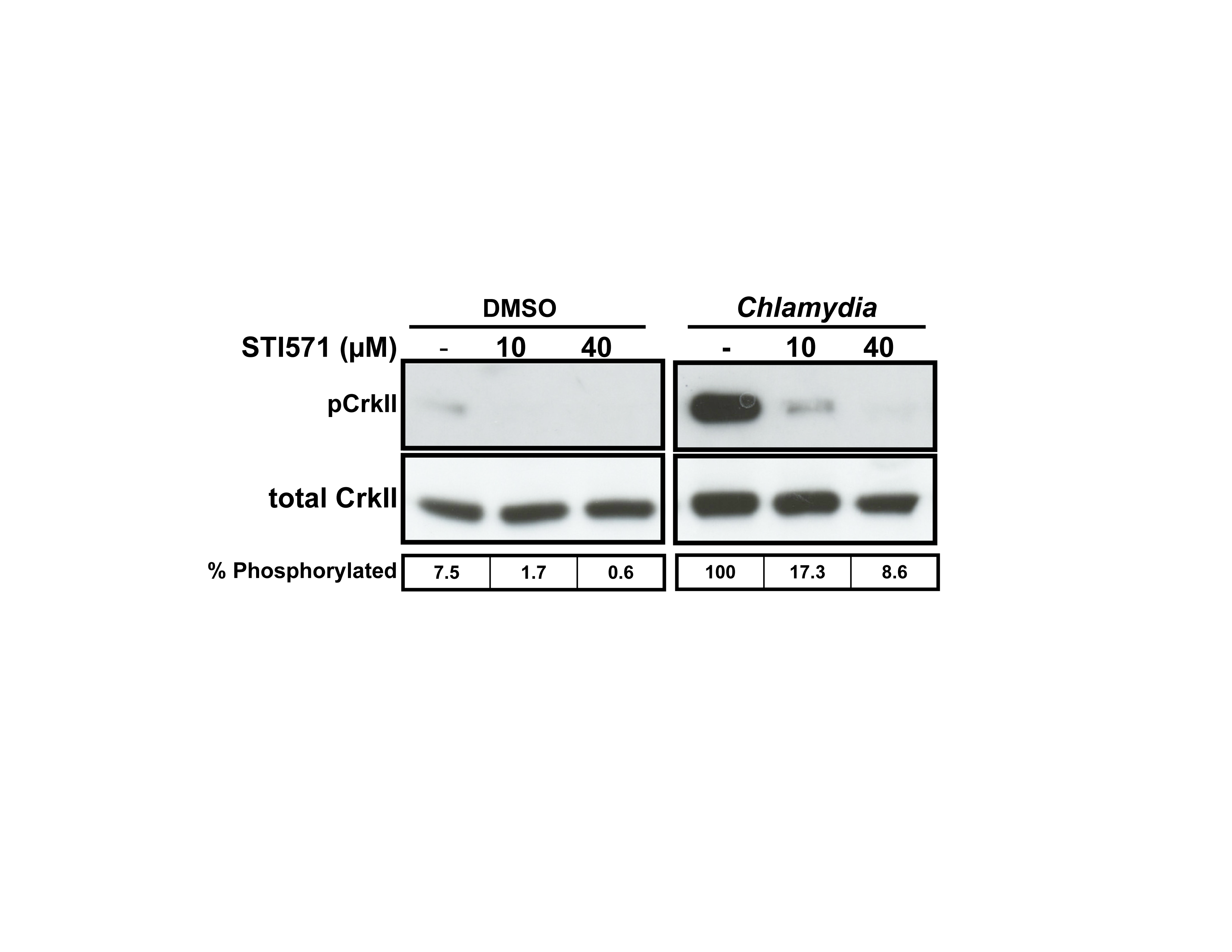

Supplement: Figure S4 — Dose-dependent inhibition of Abl kinase activity by STI571 treatment. HeLa cells were pretreated with DMSO or the indicated concentration of STI571 for 1 hr and subsequently infected with C. trachomatis in the presence of DMSO or STI571. Abl kinase activity was assessed by analyzing the phosphorylation of CrkII, an Abl kinase substrate. CrkII was immunoprecipitated from lysates and immunoblotted with anti-phospho-CrkII (Tyr221) antibody to assess phosphorylation. Blots were reprobed with total CrkII antibody to determine total protein amounts. All samples were run on the same gel and exposed the same amount of time. The percentage of phosphorylated protein compared to total protein was quantified by densitometry analysis and normalized relative to C. trachomatis-infected samples. Immunoblots shown are representative of three independent experiments. Abl kinase activity is inhibited with increasing doses of STI571. (2.79 MB TIF) [file ppat.1000021.s005.tif]

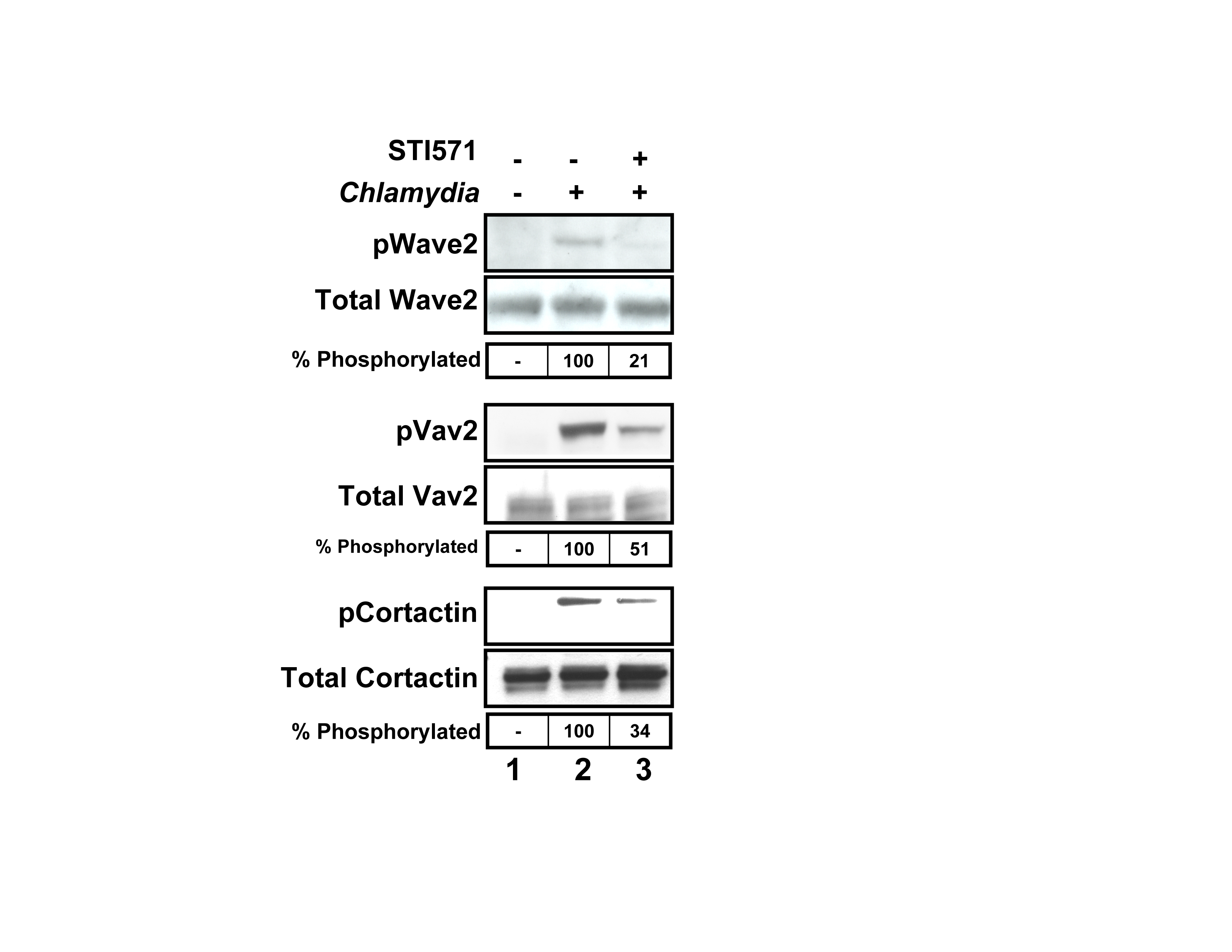

Supplement: Figure S5 — C. trachomatis -induced phosphorylation of WAVE2, Vav2, and Cortactin is diminished by STI571. HeLa cells were treated with DMSO or STI571 for 1 hour, and then subsequently infected with C. trachomatis for 1 hour. WAVE2 and Cortactin were immunoprecipitated from lysates and immunoblotted with 4G10 to assess phosphorylation. Blots were reprobed with the indicated antibody to determine total protein amounts. Lysates from the same set of samples were probed with an anti-pVav2 and total Vav2 antibodies. The percentage of phosphorylated protein compared to total protein was quantified by densitometry analysis and normalized relative to C. trachomatis-infected samples. Immunoblots shown are representative of three independent experiments. WAVE2, Vav2, and Cortactin phosphorylation is diminished by STI571. (2.51 MB TIF) [file ppat.1000021.s006.tif]

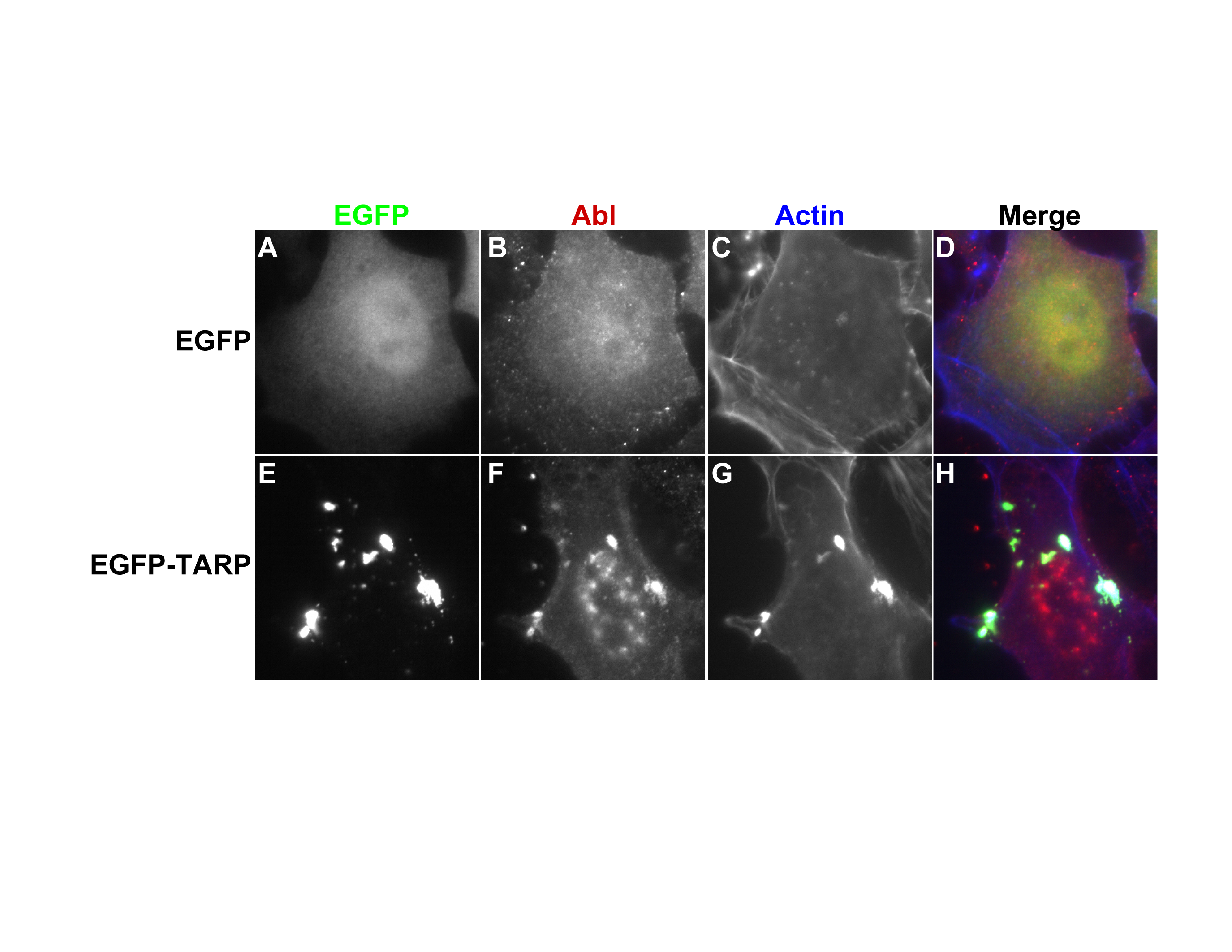

Supplement: Figure S6 — Colocalization of Abl kinase and TARP. HeLa cells were transfected with a vector encoding EGFP (A–D) or EGFP-TARP (E–H) for 24 hours. Cells were then fixed and stained with anti-Abl (B and F; red in merge). Actin was stained with phalloidin (C and G; blue in merge). The exposure time for each filter of all images was identical. Note the colocalization of EGFP-TARP and Abl kinase. (9.24 MB TIF) [file ppat.1000021.s007.tif]
